# Serum metabolomics indicates ferroptosis in patients with pantothenate kinase associated neurodegeneration

**DOI:** 10.1038/s41598-025-94838-w

**Published:** 2025-03-20

**Authors:** Beata Toczylowska, Marta Skowronska, Iwona Kurkowska-Jastrzebska, Anna Ruszczynska, Elzbieta Zieminska

**Affiliations:** 1 Nalecz Institute of Biocybernetics and Biomedical Engineering, PAS, Ks. Trojdena 4 St., 02-109 Warsaw, Poland; 2 2nd Department of Neurology, Institute of Psychiatry and Neurology, Sobieskiego 9 St., 02-957 Warsaw, Poland; 3 Faculty of Chemistry, Biological and Chemical Research Centre, University of Warsaw, Zwirki I Wigury 101 St., 02-089 Warsaw, Poland; 4 Mossakowski Medical Research Institute, PAS, A. Pawinskiego 5 St., 02-106 Warsaw, Poland

**Keywords:** PKAN patients, Metabolomics, Metallomics, Ferroptosis, Citrate, Diseases of the nervous system, Molecular neuroscience, Diagnostic markers, Lipidomics, Metabolomics

## Abstract

The core syndrome among NBIA disorders is pantothenate kinase-associated neurodegeneration (PKAN), an autosomal recessive disorder caused by mutations in the PANK2 gene. There is no therapy for PKAN; only symptomatic treatment is available. Our work aimed to identify the mechanisms induced by biochemical disturbances in the cell cycle and identify potential pharmacological targets to improve patient quality of life. Mass spectrometry (MS) (metals) and NMR spectroscopy (hydrophilic and hydrophobic compounds) were used for profile analyses of the sera of 12 PKAN patients and 12 controls to study the compounds involved in PKAN pathomechanisms. We performed ANOVA and multivariate analysis using orthogonal partial least squares discriminant analysis. We have shown for the first time that patients have 100–500-fold greater serum citrate levels than controls do, which may contribute to Fe transport and ferroptosis. Ferroptosis may be indicated by disturbances in the levels of many metals, oxidative stress, disturbances in energy production and neurotransmission or dysfunction of biological membranes. Our findings suggest that ferroptosis could be a primary cause of cell death in PKAN patients. This could be indicated by serum metabolomics.

**Supplementary Information:**

The online version contains supplementary material available at 10.1038/s41598-025-94838-w.

## Introduction

Neurodegeneration with brain iron accumulation (NBIA) comprises a heterogeneous group of progressive neurodegenerative disorders that present with progressive extrapyramidal syndrome and excessive iron deposition in the basal ganglia, mainly the globus pallidus^1,2^. The core syndrome among NBIA disorders is pantothenate kinase-associated neurodegeneration (PKAN, formerly known as Hallervorden-Spatz disease), which accounts for approximately half of NBIA cases^3^. PKAN is an autosomal recessive disorder caused by a mutation in the *PANK2* gene on chromosome 20p, the prevalence of which is estimated to be 1:1,000,000^4^. In classic PKAN, the age of onset is early, and symptoms are mainly extrapyramidal, whereas patients with a later onset often exhibit atypical clinical features. The onset of symptoms in classic PKAN usually occurs before six years of age, and the patient has a gait disorder due to dystonia and leg rigidity. The phenotype is pyramidal (spasticity, hyperreflexia, extensor toes) and extrapyramidal with prominent dystonia. Prominent oromandibular involvement is a characteristic sign^5^. Patients with late-onset atypical PKAN (in their 20 s and 30 s) have also been reported. The core symptoms might be misleading and cause diagnostic delays. Symptoms such as cognitive decline and psychiatric features or movement dysfunction mild with unilateral dystonic tremor or focal arm dystonia are described. Compared with the classical form, motor involvement tends to be less severe. However, the pathophysiology of the varied phenotypic spectra of young- versus late-onset patients remains unclear^6–8^. The role of brain iron accumulation in NBIA also remains unclear. It is becoming increasingly evident that many neurodegenerative diseases are associated with metabolic dysfunction^9^. PKAN is caused by an error in vitamin B5/pantothenate metabolism. Pantothenate kinase catalyzes the ATP-dependent conversion of pantothenate to 4-phosphopantothenate, the first committed step in coenzyme A (CoA) biosynthesis. Under normal conditions, 4-phosphopantothenate condenses with cysteine in the second step of the pathway. Decarboxylation, conjugation to an adenosyl group and subsequent phosphorylation lead to the synthesis of CoA. This high-energy carrier of acetyl and fatty acid (FA) groups is important in metabolic pathways, including the citric acid cycle (TCA); FA synthesis and oxidation; and the synthesis of cholesterol, phospholipids (PLs) and sphingolipids^9^. Currently, there is no therapy for PKAN; only symptomatic treatment is available. This study aimed to analyze the metabolic profiles of hydrophilic and hydrophobic compounds, as well as a range of metals. These investigations sought to identify potential disease biomarkers, detect biochemical disturbances in cellular processes, and probably pinpoint pharmacological targets to improve patient quality of life. We believe that metabolomics can be utilized to monitor the effects of new therapies, in patients with PKAN.

## Results

The patients’ demographic data are presented in Table 1. Statistical analysis revealed no significant differences between sexes in the control and study groups (2 × 2 contingency table, Fisher test, *P* = 1). No significant differences were observed between the ages of the patients in the study (26.1 ± 4.5 years) and those in the control group (26.1 ± 3.3) (t test,  *P* = 1).

**Table 1:** Demographic data of PKAN patients according to subgroup division and citrate levels calculated from NMR spectra.

Nr	Group	Gender	Mutation	PKAN DRS I (0–4)	PKAN DRS II (0–4)	PKAN DRS III (0–35)	PKAN DRS IV (0–28)	PKAN DRS V (0–36)	PKAN DRS VI (0–24)	DBS year	Citrate [mM]
1	P1	M	c.[573delC] + [1274 T > C]; p.[Ser191ArgfsX13] + [Leu425Pro]	3	0	27	22	24	12	2008	3.9
2	P1	M	c. [573delC] + [1583C > T]; p. [Ser191ArgfsX13] + [Thr528Met]	2	1	20	10	21	7		5.2
3	P3	F	c. [573delC] + [911-913delTCT]; p. [Ser191ArgfsX13] + [F304del]	2	0	27	25	30	11	2008	11.7
4	P3	F	c.[793G > A] + [1203delC]; p.[Asp265Asn] + [Asp403IlefsX47]; c.[377G > C] + [377G > C]; p.[Gly126Ala] + [Gly126Ala]—non pat	1	1	27	20	30	13	2009	11.9
5	P3	F	c.[1561G > A] + [del exons 3 and 4]; p. [Gly521Arg] + x	2	0	31	16	28	16	2009	10.9
6	P3	F	c.[573delC] + [863C > G]; p.[Ser191ArgfsX13] + [Pro288Arg]	3	0	24	22	32	13		12.1
7	P3	F	c. [1583C > T] + [1561G > A]; p. [Thr528Met] + [Gly521Arg]	2	2	27	17	27	8	2017	12.3
8	P2	M	c.[1561G > A] + [1561G > A]; p. [Gly521Arg] + [Gly521Arg]	4	1	34	16	36	16	2014	15.9
9	P2	M	c.[1561G > A] + [1561G > A]; p. [Gly521Arg] + [Gly521Arg]	3	0	29	17	34	14	2020	10.1
10	P3	F	c. [573delC] + [1561G > A]; p. [Ser191ArgfsX13] + [Gly521Arg]	1	3	17	21	19	8	2009	12.9
11	P3	F	c. [1583C > T] + [1561G > A]; p. [Thr528Met] + [Gly521Arg]; c.[377G > C] + [377G > C]; p.[Gly126Ala] + [Gly126Ala]—non pat	2	2	17	14	21	6	2011	12.7
12	P1	F	c.[573delC] + [863C > G]; p.[Ser191ArgfsX13] + [Pro288Arg]	2	2	29	18	21	6		0.03

The mean citrate concentration for the controls was 0.06 ± 0.03 mM.

**Fig. 1:**
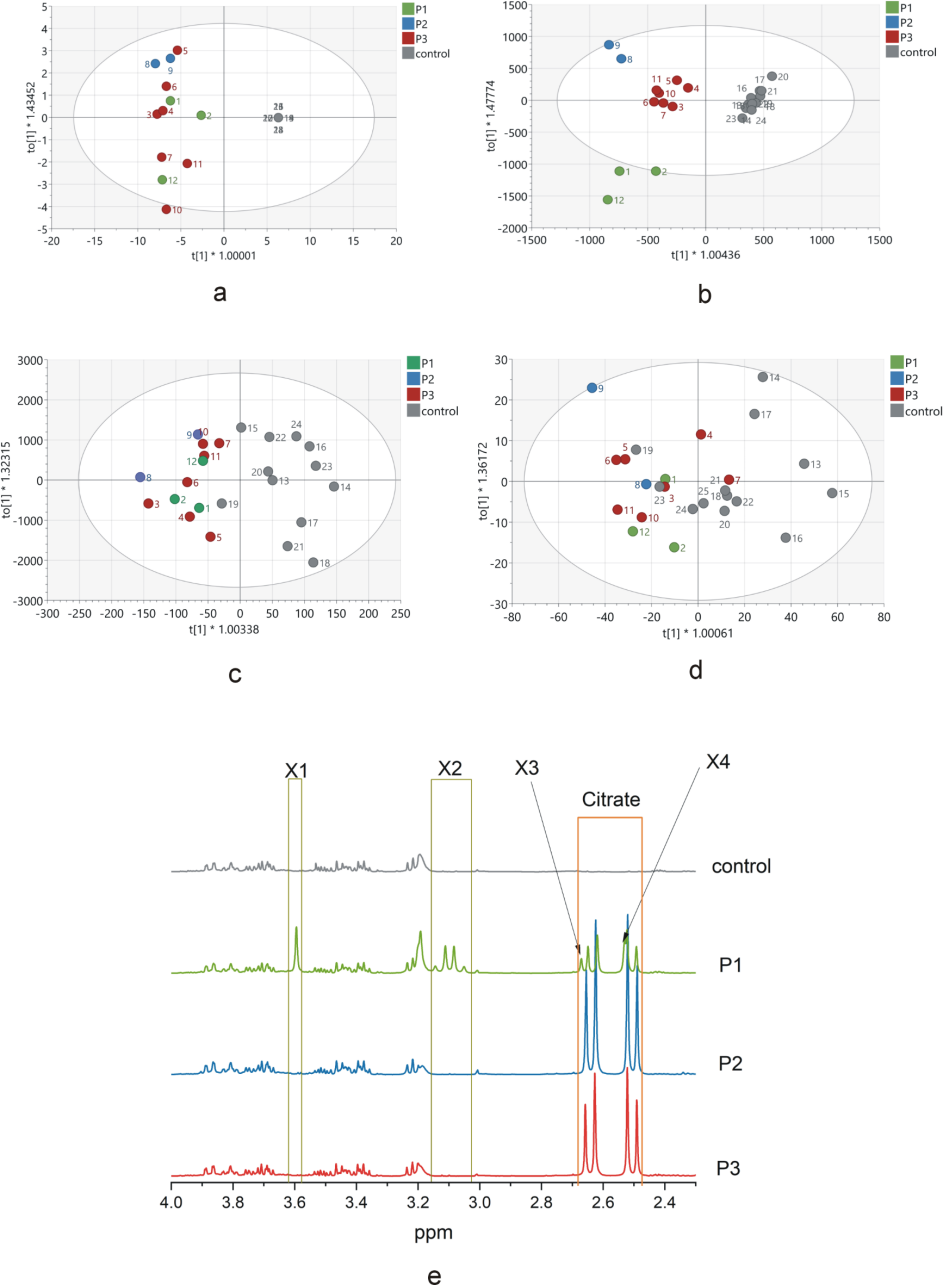
Score plot of the two-component OPLS-DA model for (**a**) clinical parameters, (**b**) serum hydrophilic compounds, (**c**) serum hydrophobic compounds, and (**d**) the serum metal panel. (**e**) Part of the typical serum NMR spectra of the hydrophilic compounds were normalized to the 1 mM TSP reference signal intensity. P1-P3 are subgroup assignments according to the OPLS-DA results. The numbers on the a-d panels represent the patient numbers (Table 1) and controls. t [1] on the abscise axis represents between-class variation in the first predictive component. to [1] on the ordinate axis represents within-class variation in the first orthogonal component. The ellipse represents the Hotelling T2 with a 95% confidence interval.

Univariate and multivariate statistical analyses (MVA) were performed for all the data, including clinical data, metal panel data, and hydrophilic and hydrophobic compound data. The results are presented in Tables 1, 2, 3 and 4 and Tables 1 and 2 SM and Fig. 1a–d.

The OPLS-DA of patient clinical data is presented in Table 1, and for controls, all the values were zero, allowing the construction of a valid model (CV-ANOVA, p < 0.001) that differentiated the groups (Fig. 1a). The model consisted of two components, one predictive and one orthogonal. For the model, the calculated cumulative R^2^ was 0.972, and the cumulative Q^2^ was 0.965. The main compounds differentiating the groups were PKAN DRS V, III, and IV (VIP values > 1). This model did not allow us to distinguish subgroups in the patient group. All patients and controls were classified correctly into their group (Fisher test, *p* < 0.0001).

MVA of the hydrophilic compounds allowed us to construct a valid model (CV-ANOVA, *p* < 0.001) that differentiated the study and control groups and additionally allowed us to identify subgroup marks as colors and names P1 to P3 (Fig. 1b). The model consisted of two components, one predictive and one orthogonal. For the model, the calculated cumulative R^2^ was 0.711, and the cumulative Q^2^ was 0.821. The compounds that most differentiated patients from controls and between the PKAN subgroups of patients and controls are presented in Table 2 with ANOVA *p* values and VIP values and Table 1 SM, respectively. All patients and controls were classified correctly into their group (Fisher test, *p* < 0.0001).

**Table 2:** Statistical analyses of hydrophilic compounds.

Compound	PKAN vs control [%]	PKAN/Controls [a.u.]	*P* value	VIP value
Formate	283	1840 (1737–2019)/519.5 (375.25–1202)	**0.001**	
Histidine	74	1801.194 ± 660.245/2425.750 ± 807.624	**0.05**	
Phenylalanine	124	558(494.5–607.25)/410.5(346.75–51-)	**0.013**	
Tyrosine	131	1290.111 ± 302.218/987.167 ± 180.755	**0.007**	
Urea	103	9152.000 ± 2890.810/8857.167 ± 4605.857	0.853	
Cis-aconitate	294,000,000	284.5 (0.0–506.0)/0.0 (0.0–0.0)	**0.001**	
α-D glucose	93	29,840.444 ± 5576.953/32,079.583 ± 7103.194	0.400	
Unassigned 4.03 ppm	77	1840.111 ± 272.109/2390.833 ± 652.439	**0.013**	
X1 (3.61 ppm)	1,770,000,000	0.0 (0.0–131,794.5)/0.0 (0.0–0.0)	0.071	2.54
Myo-inositol	79	3443.815 ± 886.884/4375.250 ± 874.265	**0.027**	
Glycine	48	14,659.5(13,676.5–20,443.25)/33,297.0 (26,901.25- 36,688.25)	**0.001**	
D-Glucose	90	36,178.639 ± 5189.097/40,356.500 ± 8670.686)	0.166	
Glycerophosphocholine/Choline	196	45,169.5 (37,670.0–133,138.25)/56,697.0 (32,271.0–75,935.0)	0.817	
9-Methyluric acid	124	2903.926 ± 1417.733/2351.250 ± 1029.35	0.313	
Malonic acid	499	3065.0(2589.03258.5)/595.0(463.75–714)	**0.001**	
X2 (3.12 ppm)	256,880,000	0.0 (0.0–74,996.25)/0.0 (0.0–0.0)	0.071	1.21
L-cysteine	158	584.0(414.01225.333)/532.5(392.5–702.25)	0.286	
Creatine/Creatinine	119	7378.5(4994.0–9912.0)/7093.0(5736.25–7896.5)	0.525	
X3 (2.70 ppm)	1,428,400,000	0.0 (0.0–40,086.75)/0.0 (0.0–0.0)	0.071	
Citrate	15,276	347,273.0(182,821.5 420,493.25)/2413.5(1413.0–2785.75)	**0.001**	5.06
X4 (2.56 ppm)	31,200,000	0.0 (0.0–91,905.75)/0.0 (0.0–0.0)	0.071	1.35
L-Glutamine	85	5678.5(4762.56571.0)/7594.0(4075.25–9131.75)	0.248	
Pyruvate	111	3128.667(2380.254219.5)/3544.0(2088.0–4030.25)	0.708	
L-Glutamate	42	1221.0(899.75–1847.25)/2615.5(2192.0–4920.75)	**0.001**	
Acetone	282	11,773.667(9741.5–21,212.5)/5670.5 (4578.75–8036.5)	**0.001**	
Methionine	88	6489.556 ± 1522.374/7365.917 ± 3061.081	0.384	
N-Acetyl glycoprotein	84	30,239.25 ± 4186.162/36,183.583 ± 7342.933	**0.023**	
Acetate	92	2869.0(2049.5–4452.75)/3009.0(1517.75–6429.5)	0.954	
GABA	68	714.111 ± 314.885/1044.5 ± 274.839	**0.012**	
Lysine	88	1287.0(1039.25–1712.25)/1421.0(1296.25–1900.75)	0.083	
Lipids (VLDL)	39	931.5(140.5–1526.5)/1968.0(846.25–2777.25)	0.056	
Alanine	67	16,187.556 ± 6258.24/24,099.5 ± 4135.716	**0.001**	
Lactate	107	60,215.528 ± 33,948.999/56,181.167 ± 16,287.477	0.714	
Lipids CH_2_ (LDL and VLDL)	60	43,634.5(35,389.25–67,367.25)/76,919.5(33,136.25–96,782.5)	0.204	
3-OH butyrate	84	2832.167(414.75–10,445.250/535.5(343.0–13,882.5)	0.862	
Valine	81	8155.556 ± 1826.395/10,068.833 ± 1853.672	**0.018**	
Isoleucine	98	1546.5(1390.75–2309.0)/1734.0(1520.5–2345.25)	0.488	
Leucine	102	2785.0(2426.75–3325.417)/2604.5(2354.0–3728.0)	0.729	
Lipids CH_3_ (LDL,VLDL,HDL)	74	35,984.0(31,781.25–40,608.333)/52,325.0(24,456.5–76,755.0)	0.166	

The data are presented as changes in PKAN patient compound levels vs control levels and are expressed as percentages; for PKAN patients and controls, the means ± SDs or medians (25%-75%) (depending on the ANOVA used) are expressed in arbitrary units (a.u.), and *p* values and VIP values from the MVA analysis are presented. The bold symbols indicate significant *p* values.

According to our results, we distinguished three subgroups in patient groups P1 to P3 (Fig. 1b). Subgroup P1 differed from the other two subgroups in the presence of a high concentration of unidentified compound(s), which was absent in the other patients and controls. Additionally, the citrate level was lower than that in other patients and, in one patient, lower than that in controls (Table 2). Other metabolite differences are presented in Table 4 (unassigned signal at 4.03 ppm was present in all subjects). Subgroup P2 was the homozygous subgroup, and patients in this subgroup had the highest citrate level. Typical spectra of each subgroup and control are presented in Fig. 1e, which shows the signals of citrate and unidentified compounds X 1–4. The spectra were normalized to the intensity of the 1 mM TSP reference standard.

OPLS-DA of lipid compounds allowed us to construct a valid model (CV-ANOVA, *p* = 0.026) for differentiating the study and control groups (Fig. 1c). The model consisted of four components, one predictive and three orthogonal. For the model, the calculated cumulative R^2^ was 0.99, and the cumulative Q^2^ was 0.729. This model does not allow us to identify subgroups as in the previous model. The compounds that most differentiated patients from controls are presented in Table 3—ANOVA *p* values and VIP values and PKAN subgroups from controls in Table 6. A total of 83% of the patients were classified correctly in 100% of their groups, and 83% were classified correctly in the control group (Fisher test, *p* < 0.0001).

**Table 3:** Statistical analyses of hydrophobic compounds/functional groups.

Compound/functional group	PKAN vs control	PKAN/Controls [a.u.]	*P* value	VIP value
Estriol	61	237.972 ± 101.072/389.452 ± 179.907	**0.019**	
Estrone	117	275.861 ± 134.484/236.701 ± 186.832	0.562	
Testosterone	138	135.318(86.69–281.75)/124.07(59.623–211.0	0.326	
Phosphatidylcholine Phosphatidylethanolamine Sphingomyelin PUFAs and MUFAs	94	38,923.052 ± 20,817.804/41,465.251 ± 31,150.534	0.816	
1,2-DAG, 2-MG	104	711.137 ± 384.187/681.901 ± 343.094	0.846	
Palmitic acid in FA	84	5585.989 ± 3090.779/6656.076 ± 4514.238	0.505	
1,2-DAG	86	3219.825(2007.683–6099.0)/3401.903(1326.718–6461.75)	0.908	
Triglycerides	105	4763.691(2437.998–7445.0)/3714.561(2808.943–6287.75)	0.686	
1,3-DAG	134	4579.078(1933.529–5536.75)/3521.119(0.0–5595.953)	0.441	
1-MAG	142	4549.404(1911.978–5439.25)/3152.307(0.0–5282.091)	0.271	
Phosphatidylcholine	82	57,786.119(37,158.434–102,456.75)/41,284.5(16,771.002–106,831.5)	0.603	
Sphingomyelin	81	24,855.816 ± 14,311.044/30,563.629 ± 23,947.893	0.497	
Phosphatidylethanolamine	982	1046.895(700.0–1855.5)/0.0(0.0–86.69)	**0.001**	
PUFAs	111	12,442.828(5356.867–16,536.5)/8200.223(3475.186–18,009.5)	0.453	
Linoleic acid	81	16,146.041(10,131.893–27,927.5)/18,952.162(8184.33–39,436.0)	0.817	
Palmitic acid	83	21,810.304 ± 12,631.189/26,318.516 ± 18,224.045	0.489	1.13
Hexanoylglycine	120	7995.733(4323.736–12,815.621)/6010.304(2714.705–11,165.75)	0.248	1.24
FAs	90	29,897.072 ± 16,579.295/33,115.222 ± 30,508.415	0.751	2.05
Vaccenic acid	86	5199.258 ± 3046.482/6017.577 ± 4312.12	0.597	
Palmitoleic acid	115	3962.431 ± 1839.310/3456.877 ± 2132.503	0.540	
1,3-DAG, 1-MAG	88	32,428.971 ± 16,853.284/36,853.667 ± 25,556.377	0.622	1.03
Saturated FAs, PUFAs and MUFAs	91	184,782.746 ± 98,644.575/203,606.522 ± 136,950.476	0.703	2.16
Saturated FAs and PUFAs	96	607,180.270 ± 319,346.332/635,208.583 ± 428,559.252	0.858	2.98
Cholesterol esters	84	103,561.890 ± 59,626.443/123,857.748 ± 81,462.580	0.493	2.27
Free cholesterol	90	51,152.356 ± 30,122.303/56,551.223 ± 38,554.376	0.706	1.14
Saturated FAs, PUFAs and MUFAs	93	163,269.112 ± 86,449.441/176,283.381 ± 112,082.496	0.753	1.69
Saturated FAs	96	29,293.678 ± 13,213.132/30,528.094 ± 18,776.590	0.854	
24S–Hydroxycholesterol	102	570.317 ± 313.865/560.214 ± 306.391	0.937	
Free cholesterol and cholesterol esters	85	129,731.878 ± 75,499.335/152,139.821 ± 102,243.407	0.548	2.37
7-Lathosterol	66	72.796(29.574–240.895)/139.0(82.791–300.685)	0.119	

The data are presented as changes in the compound/functional group of PKAN patients vs the control group; the data are expressed as percentages; for PKAN patients and controls, the means ± SDs or medians (25%-75%) (depending on the ANOVA used) are expressed in arbitrary units (a.u.), and *p* values and VIP values from the MVA analysis are presented. The bold symbols indicate significant *p* values.

For the metal panel OPLS-DA, a valid model (CV-ANOVA, *p* = 0.006) was constructed to differentiate between the patient and control groups (Fig. 1d). The model consisted of two components, one predictive and one orthogonal. For the model calculated, the cumulative R^2^ was 0.972, and the cumulative Q^2^ was 0.53. Additionally, this model did not allow the identification of subgroups. The metals that most differentiated patients from controls are presented in Table 4. The ANOVA *p* values and VIP values and differences between subgroups are shown in Table 3 SM. A total of 92% of the patients were classified correctly into their group, and 77% of the controls were classified correctly (Fisher test, *p* = 0.0008).

**Table 4:** Statistical analyses of metals.

Metal	PKAN vs control [%]	PKAN/Controls [μM]	*P* value	VIP value
Mg	88	623.020 ± 78.678/706.628 ± 72.401	**0.011**	1.05
K	78	3041.516 ± 427.508/3910.986 ± 590.055	**0.001**	3.52
Ca	91	949.798 ± 104.017/1041.860 ± 119.623	0.052	1.16
Cr	89	5.620 ± 0.866/6.337 ± 0.716	**0.033**	
Fe	43	7.627(3.655–11.948)/20.886(15.539–24.824)	**0.003**	
Co	148	0.0141(0.0116–0.0201)/0.00904(0.00791–0.0133)	**0.009**	
Ni	105	0.1(0.081–0.113)/0.0793(0.0629–0.117)	0.446	
Cu	98	13.588 ± 2.484/13.915 ± 2.496	0.746	
Zn	55	3.7(2.895–4.369)/5.936(5.39–9.787)	**0.001**	
Al	70	5.749(2.432–11.596)/7.200(5.712–11.374)	0.277	
B	339	6.946(4.236–13.244)/2.396(1.436–3.378)	**0.001**	
Cd	76	0.000477(0.000407–0.00123)/0.000684(0.000373–0.00143)	0.786	
Se	74	0.876 ± 0.177/1.183 ± 0.125	**0.001**	
Mn	203	0.377(0.274–0.47)/0.0319(0.0237–0.04)	**0.003**	
Sr	73	0.284(0.260–0.335)/0.380(0.313–0.501)	**0.011**	

The data are presented as changes in PKAN patient metal levels vs control levels expressed as percentages; for PKAN patients and controls, the means ± SDs or medians (25–75%) (depending on the ANOVA used) are expressed in μM, and *p* values and VIP values from the MVA analysis are presented. The bold symbols indicate significant p values.

PKAN patients have different metabolite profiles, and the differences were assigned, according to the statistical parameter (VIP value), as unidentified compound(s) with NMR signals X1 and X4 and citrate, palmitic acid, hexanoylglycine, saturated FA, PUFA and MUFA, 1,3-DAG, 1-MAG, free cholesterol and cholesterol esters (Tables 2 and 3). Additionally, the metal levels differed among the PKAN patients (Table 4), and the Mg, K, and Ca (VIP) levels differed among the groups. All the analyzed metal concentrations, except those of Ca, Ni, Cu, Al and Cd, were significantly different.

On the basis of the results of univariate and MVA analyses, we present metabolite differences in the metabolite levels of the PKAN patient group versus controls (direction of changes) and probable mechanisms induced by these compounds (Table 5). Analysis of statistically significant and VIP metabolites in metabolic pathways was performed using the Kyoto Encyclopedia of Genes and Genomes (KEGG)^10^.

**Table 5:** Functions and metabolic pathways associated with the metabolites (VIP > 1 or *p* < 0.05) in PKAN patients; the directions of changes found in each of the models compared with the control are presented.

Metabolites	Metabolic pathway	Most likely pathomechanism	PKAN vs control
Estriol	Steroid hormone biosynthesis	Disturbances in energy production and neurotransmission	↓
Saturated fatty acids (FAs), Monounsaturated FAs (MUFAs), Polyunsaturated FAs (PUFAs)	FA biosynthesis and metabolism	Disturbances in energy production and cell membrane function Ferroptosis	↓
Palmitic acid			↓
Cholesterol	Cholesterol metabolism	Disturbances in neurotransmission	↓
Cholesterol ester			↓
Phosphatidylethanolamine	Glycerophospholipid metabolism	Disturbances in cell membrane function Ferroptosis	↑
Glycine	Purine metabolism Glycine, serine metabolism	Disturbances in neurotransmission Oxidative stress	↓
Malonic acid	Pyrimidine, pyruvate and beta alanine metabolism, FA biosynthesis		↑
Histidine	Histidine metabolism Glutathione/Cysteine and methionine metabolism	Disturbances in neurotransmission Oxidative stress	↓
Glutamate			↓
Methylamine	Carbone metabolism	Disturbances in energy production	↑
GABA	Arginine and proline metabolism	Disturbances in neurotransmission Oxidative stress	↓
Creatinine			↓
Alanine	Taurine, alanine, aspartate, glutamate and pyruvate metabolism, TCA cycle	Disturbances in neurotransmission Oxidative stress Disturbances in energy production	↓
Lactate			↓
Formate			↑
Citrate			↑
Cis-aconitate			↑
Myo-inositol	Inositol metabolism	Disturbances in energy production	↓
Acetone	Synthesis and degradation of ketone bodies	Disturbances in energy production	↑
Valine	Valine, leucine, isoleucine degradation, Purine metabolism, Glycine, serine and threonine metabolism	Disturbances in neurotransmission Oxidative stress	↓
Phenylalanine	Phenylalanine metabolism Tyrosine, phenylalanine and tryptophan biosynthesis		↑
Tyrosine			↑
Dimethylurea	Caffeine metabolism	Intestinal malabsorption disorders	↑
N-acetylglycoprotein	different pathways	Inflammation marker	↓

↑↓—level values increased or decreased compared with those of the control.

**Fig. 2:**
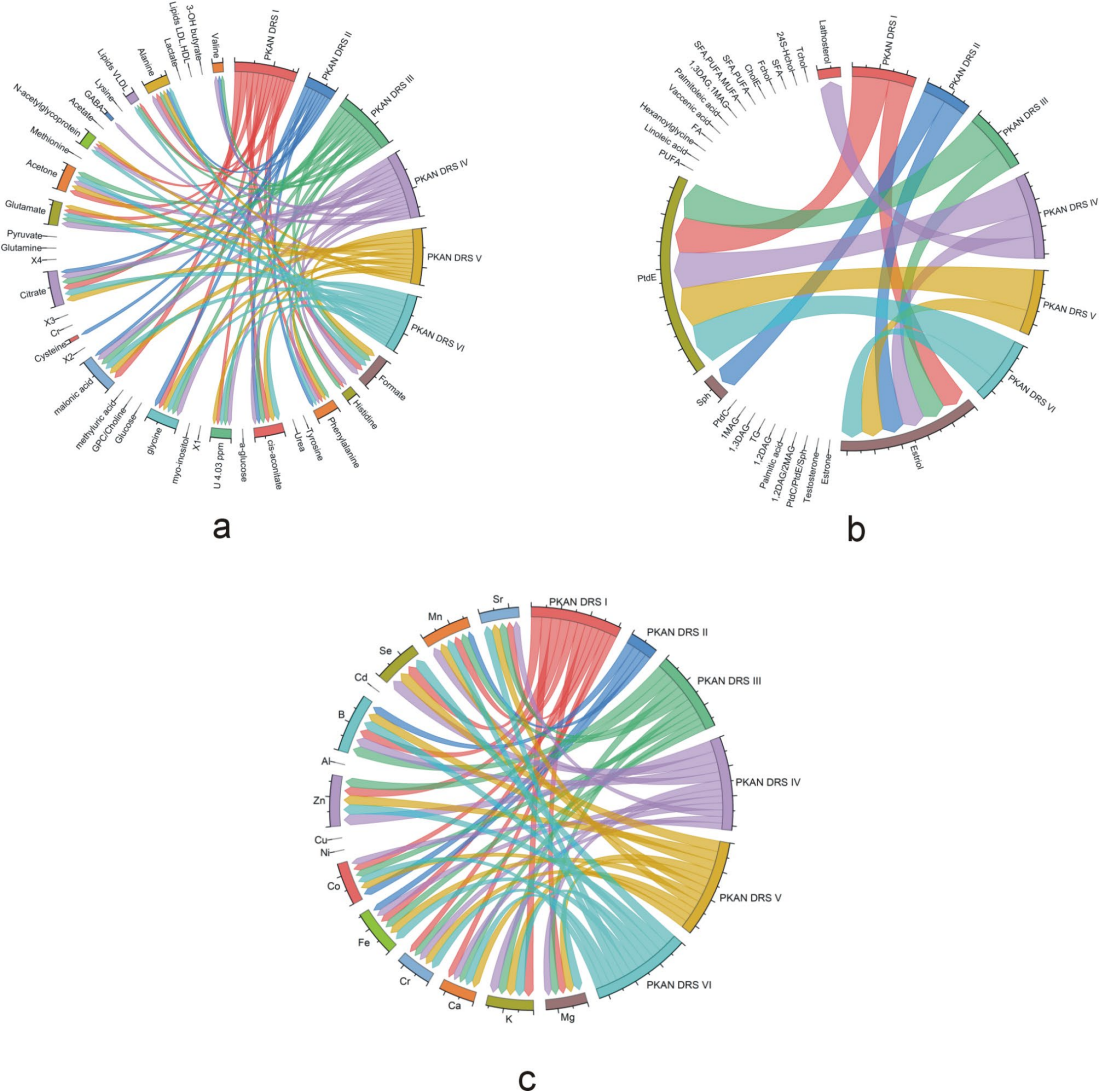
Correlation (Spearman rank correlation) chord diagram of the clinical parameters PKAN DRS I-VI and hydrophilic compounds (**a**), hydrophobic compounds (**b**) and metals (**c**). Only significant correlation coefficients are presented. TChol—total cholesterol (free and ester), HChol—hydroxycholesterol, FChol—free cholesterol, EChol—cholesterol ester, SFA—saturated fatty acids, PUFA—polyunsaturated fatty acids, MUFA—monounsaturated fatty acids, DAG—diacylglycerol, MAG—monoacylglycerol, FA—fatty acids, PtdE—phosphatidylethanolamine, PtdC—phosphatidylcholine, Sph—sphingomyelin, TG—triglycerides, GPC—glycerophosphorylcholine, X1-X4—unassigned signals (Table 2).

To date, the patient’s condition has been assessed by clinical parameters according to the PKAN DRS. We examined the relationships between these parameters, serum biochemical changes, and changes in metal levels. The results of the correlation analyses are presented in Fig. 2a–c. In the chord diagrams, we did not differentiate between the directions of the correlations, so they are shown in Table 6. Metabolites that are positively correlated with clinical parameters are associated with oxidative stress and disturbances in cell membrane function, e.g., ferroptosis, and are negatively correlated with neurotransmission and energy production.

**Table 6:** Directions of statistically significant correlations.

DRS scale	Description	Significant positive correlation	Significant negative correlation
PKAN DRS I	Cognition	Formate	Histidine
		Phenylalanine	U 4.03 ppm
		Cis-aconitate	Glycine
		Malonic acid	Glutamate
		Cysteine	N-acetylglycoprotein
		Citrate	Lipids (VLDL)
		Phosphatidylethanolamine (PtdE)	Alanine
		Co, B, Mn	Estriol
			Mg, K, Ca, Cr, Fe, Zn, Al, Se, Sr
PKAN DRS II	Behavior	Phenylalanine	Glycine
		Cis-aconitate	Alanine
		Malonic acid	Valine
		Cysteine	Estriol
		Citrate	Sphingomyelin
		Co, B, Mn	Fe
PKAN DRS III	Disability	Formate	Histidine
		Phenylalanine	U 4.03 ppm
		Cis-aconitate	Glycine
		Malonic acid	Glutamate
		Citrate	Lipids (VLDL)
		Acetone	Alanine
		Phosphatidylethanolamine (PtdE)	Valine
		Co, B, Mn	Estriol
			Mg, K, Ca, Cr, Fe, Zn, Al, Sr
PKAN DRS IV	Parkinsonism	Formate	Histidine
		Phenylalanine	U 4.03 ppm
		Cis-aconitate	Glycine
		Malonic acid	Glutamate
		Citrate	N-acetylglycoprotein
		Acetone	GABA
		Phosphatidylethanolamine (PtdE)	Alanine
		Co, B, Mn	Valine
			Estriol
			Lathosterol
			Mg, K, Ca, Cr, Fe, Zn, Al, Se, Sr
PKAN DRS V	Dystonia	Formate	U 4.03 ppm
		Phenylalanine	Glycine
		Cis-aconitate	Glutamate
		Malonic acid	N-acetylglycoprotein
		Citrate	Alanine
		Acetone	Estriol
		Phosphatidylethanolamine (PtdE)	Mg, K, Ca, Cr, Fe, Zn, Al, Se, Sr
		Co, B, Mn	
PKAN DRS VI	Other symptoms: Speech, chorea, spasticity	Formate	U 4.03 ppm
		Phenylalanine	Glycine
		Cis-aconitate	Glutamate
		Malonic acid	N-acetylglycoprotein
		Citrate	Alanine
		Acetone	Lipids (VLDL)
		Phosphatidylethanolamine (PtdE)	Estriol
		Co, B, Mn	Mg, K, Ca, Cr, Fe, Zn, Al, Se, Sr

### Supplementary materials

Three tables (Tables 1, 2 and 3 SM1) showing the significant differences between the PKAN subgroups of hydrophilic and hydrophobic compounds and metals. The data are presented as the mean ± SD or median (25–75%)—depending on the test used for the PKAN patient subgroup vs control levels and between PKAN subgroups. The results of the analysis of correlation–correlation coefficients are presented in the Excel file (SM2). Significant correlation coefficients are in red.

## Discussion

We considered the group of patients as a single group because of the small number of patients in each predefined subgroup. We analyzed serum metabolite changes in patients with genetic diseases. From the analysis of the obtained results (Tables 2, 3 and 4), we can conclude that these patients have disturbances in cycles triggered primarily by mechanisms related to oxidative stress, energy disorders, neurotransmission, or dysfunction of biological membranes. Metabolites participate in multiple metabolic cycles. Thus, each metabolite is involved in many mechanisms (Table 5). This also refers to metals participating in many cycles. Therefore, assigning a specific element to a single mechanism is not possible. However, it must be remembered that the changes in metabolite or metal levels in the brain can be either the same or different than those measured in serum. In our previous studies^11,12^ on rats, we compared the metabolite levels measured by the same method, NMR spectroscopy, in different body fluids (serum, urine) and brain extracts. Regardless of the media studied, we noticed that disturbances often appeared in the same cycles but were related to different compounds from the cycle, and the directions of changes in metabolite levels in the brain and periphery can be consistent or opposite. A similar situation was observed when metals were measured in the serum and brain^13^. Comparing the serum metabolite levels with the in vivo NMR spectroscopy results of the brains of Parkinson’s disease patients, we did not observe consistency regarding the compounds, but it is possible to observe disturbed biochemical cycles^14^. Therefore, by measuring serum metabolite changes, we can specify disturbed cycles but not particular compounds that change in the brain. The studied disease has a genetic basis. This disease affects many organs in the body, particularly the brain, which causes most symptoms of the disease. As mentioned above, by measuring the serum metabolic profiles and metal levels, we could identify disturbance cycles and thus elucidate the mechanisms involved in the disease.

The mechanism that seems to have a prominent impact on the disease progression of PKAN patients is oxidative stress, which leads to cell death through ferroptosis. Some authors consider ferroptosis as a specific form of regulated necrosis^15^. Feng et al.^16^ described four ways to initiate ferroptosis. Ferroptosis inducers can be divided into four classes. Class 1 inducers act by depriving cells of the amino acid cysteine. These compounds act by inhibiting system Xc, a transmembrane cystine–glutamate antiporter that imports cystine (the oxidized, disulfide form of cysteine) into cells. When this process is blocked, cysteine is depleted from these cells. Cysteine has a number of functions in cells, primarily in the context of ferroptosis, as a building block for glutathione (GSH) biosynthesis. GSH is a cofactor and substrate for glutathione peroxidase (GPX4) and is essential for the lipid repair function of this enzyme. GSH depletion through cysteine starvation leads to a loss of GPX4 activity, resulting in the accumulation of unrepaired lipid peroxides and death by ferroptosis. In addition, high extracellular concentrations of the amino acid neurotransmitter glutamate can act as class 1 inducers of ferroptosis^16^. Among the various lipids, polyunsaturated fatty acids (PUFAs), which are bound to several phospholipids, such as phosphatidylethanolamine (PE) and phosphatidylcholine (PC), are responsible for lipid peroxidation-induced ferroptosis. Cells acquire essential fatty acids from blood and lymph to produce various polyunsaturated fatty acids (PUFAs) via different PUFA biosynthetic pathways^17^. Aoun et al.^18^ reported that the red blood cells of patients with PKAN have a different qualitative composition of membrane lipids than those of the control group. In our study, we observed increased levels of PEs and decreased levels of fatty acids (FAs), polyunsaturated fatty acids (PFAs), and monounsaturated fatty acids (MUFAs), indicating membrane damage and impaired lipid synthesis due to acetyl-CoA deficiency. Ferroptosis is also characterized by iron-dependent mitochondrial constriction and the accumulation of reactive oxygen species (ROS). This process is initiated by the malfunction of glutathione-dependent antioxidant defense mechanisms, leading to uncontrolled lipid peroxidation and subsequent cell death. Glutathione synthesis and its dependent enzyme, GPX4, are also inhibited^19^. This mechanism is confirmed by the observed increase in the serum PE concentration; decreased levels of FAs, polyunsaturated fatty acids, and MUFAs; and decreases in cysteine, glutamate and glutamine in our study (these findings indicate disturbances in Cys, Glu and Gln metabolism). It is not clear whether ferroptosis is induced only in brain cells (where iron accumulation is observed) or whether it affects, or also other tissues^20,21^. The brain requires high levels of iron and a constant supply of oxygen. Therefore, optimal iron levels are crucial for maintaining normal brain functions, such as myelin synthesis and the regulation of brain neurotransmitters. Dopamine, norepinephrine, serotonin, and GABA are neurotransmitters whose synthesis and metabolism depend on iron. Iron deficiency can result in reduced production of some neurotransmitters, which can negatively affect mood, cognitive, and motor functions. However, excess iron can cause neurotoxicity^22^. Our study revealed that individuals with genetic mutations have serum iron concentrations that are twice as low as those in the control group. How exactly Fe is deposited in the cells of patients with PKAN is not fully understood because there is no good animal model for research. Santambrogio et al.^23^ used an in vitro model built from hiPSC-derived cells from PEKAN patients and healthy patients, using a mixed culture of cells mainly from the striatum, medium spiny neurons and, in a small proportion, glial cells. In hiPSC-derived astrocytes, Fe accumulation in the cytosol has been described. Excess Fe triggers ferroptosis, which allows hiPSC-PKAN astrocytes to develop a stellate, reactive phenotype, thus acquiring a cytotoxic feature, confirmed by an increase in glutamate secretion resulting in reduced survival of hiPSC-neurons from PKAN patients in comparison with healthy controls in the case of coculture.

Most metals enter the body through food and are absorbed through passive transport (diffusion) from the gastrointestinal tract^24–26^ and then transported to target cells, mainly as protein-bound complexes. An examination of the metal concentrations revealed that 10 out of the 14 metals were present at lower concentrations in the affected individuals than in the control individuals (Table 4). In the analysis, we also included toxic metals from the environment. The lower metal levels could be due to the cell membranes in the intestines being structurally abnormal due to lipid peroxidation, including that of the cell membranes, which may impair their function. The results of the work of Leoni et al.^27^ indicate disorders in the transport of fats, including cholesterol and vitamins. In our studies, we also observed a reduced level of cholesterol and its derivatives/precursors (Table 3).

We observed that in the serum, the levels of Mn increased by twofold, those of Co increased by 1.5-fold, and those of B increased by 3.4-fold. These metals are essential for proper organismal function and play crucial roles in brain development^25^. These three elements positively correlate with the clinical parameters of the PKAN DRS (Fig. 2c, Table 6), which means that the higher their levels are, the greater the number of assessments on the PKAN DRS, which indicates more severe symptoms of disease or disease progression. Mn transport across the blood–brain barrier occurs through active transport involving the same transporter as Fe. There is an inverse correlation between iron uptake and manganese absorption because both compete for the same divalent metal transporter, DMT1^28^. Thus, elevated serum levels of Mn and decreased levels of Fe could cause an accumulation of Fe in the brain with a decreased level of Mn. Since no experimental or literature data exist, we could not confirm our observations or, thus, this hypothesis.

There are studies^29^ describing links between elevated levels of cobalt and the development of Parkinson’s disease because of the toxicity of cobalt above a certain level. However, it is essential for humans since it is a cofactor for cobalamin (vitamin B12). In our studies, the serum Co concentration was 1.5-fold greater than that in the controls. This could, together with other factors, influence or induce symptoms of Parkinson’s disease in PKAN patients.

Boron, however, is not a metal; it has a significant effect on metabolism, and its effects are known to be positive. It influences the activity of at least 26 enzymes (oxidoreductases, transferases, hydrolases, and isomerases) studied in various animals, plants, or cell cultures by acting directly on the enzyme, binding to cofactors (such as NAD) or substrates, or through unknown mechanisms. B is a regulator of enzymatic activity in pathways closely related to the metabolism of energy substrates, insulin release, and the immune system and reduces the incidence and severity of inflammatory diseases. It decreases oxidative stress by increasing antioxidant defense mechanisms, and another study suggested its influence on nitrogen-containing substances such as amino acids and proteins^30,31^. In our study, its levels were greater than those in controls, but further studies are needed to explain its role and increase in this disease.

In the sera of the PKAN patients, we observed a high level of citric acid (100–500 times greater than that in the control group) (Table 1). Citrate is released from bone into the plasma during bone resorption. This is the major source of citrate needed to maintain normal plasma concentrations. Dietary citrate is an important source of plasma citrate when available. Depending on the amount ingested, citrate is absorbed from the gastrointestinal tract into the hepatic portal circulation and ultimately into the systemic circulation. Citrate is a key substrate that mediates cellular energy metabolism^32^. In mitochondria, citrate is produced by the condensation of acetyl-CoA and oxaloacetate by citrate synthase. It then becomes a substrate in the tricarboxylic acid (TCA) cycle and provides the major source of cellular ATP. Cytosolic citrate is required for the de novo synthesis of fatty acids. It is derived either from mitochondrial release by a specific citrate carrier or from extracellular import by citrate transporters across the plasma membrane^33^. In addition to increased citric acid levels, the observed elevated formic acid levels in PKAN patients indicated a disrupted TCA cycle caused by mitochondrial damage in PKAN disease. Werning et al.^34^ suggested an alternative pathway for obtaining energy from the citric acid present in the serum of erythrocytes. However, this mechanism seems to be inefficient, and the circulation of citrate in the body is disrupted, leading to excess citrate in the serum. Citric acid easily binds to iron ions and can be transported to the brain. Oligodendrocytes are particularly sensitive to iron levels; thus, ferroptosis can easily lead to their damage^35^. Under laboratory conditions, iron in the form of citrate is used to induce ferroptosis in cell cultures^36,37^. Oligodendrocytes are responsible for forming myelin sheaths. According to MRI studies of the brain, the region most affected by iron accumulation is, e.g., the globus pallidus^38^, which is built from myelinated nerve fibers and is likely to accumulate iron from damaged cells.

On the basis of our research, we propose the following hypothesis to explain the progression of the disease in individuals with a mutation in the PANK2 gene: this mutation disrupts the function of the TCA cycle. If the cycle is blocked or slowed at certain steps due to a lack of CoA, intermediates upstream of the block may accumulate. Citric acid is one of these intermediates. As citrate begins to accumulate, it is subsequently released into the cytosol. A specific concentration of citrate inside the cell may be necessary to initiate ferroptosis. Owing to oxidative stress, the membranes of the mitochondria and endoplasmic reticulum become damaged (as indicated by an increase in serum phosphatidylethanolamine). Oligodendrocytes, which are particularly sensitive to the iron level needed for enzymatic reactions, are especially vulnerable. Additionally, medium spiny neurons (MSNs) in the striatum are known to be highly metabolically active, making them potentially more susceptible to mitochondrial dysfunction and oxidative stress. Studies have shown that MSNs are particularly prone to iron-induced damage in other neurodegenerative conditions^39^. The metabolic changes in the brain resulting from PANK2 mutations can also have systemic effects, influencing metabolism in other tissues and potentially impacting serum citric acid levels. An excess of unprocessed citrate likely appears in the serum via cerebrospinal fluid, although we do not yet have evidence to support this. Another source of citrate in the serum may be food, both plant-based and highly processed, as citrate is commonly used as a harmless preservative. Citrate, which readily binds to metals, particularly iron, can then be transported to the brain in a manner similar to how aluminum citrate is transported. Nagasawa et al.^40^ suggested the involvement of the Xc system (a glutamate/cystine antiporter) in their studies. Ferroptosis is induced in cell cultures by iron bound to citrate, and it is believed that this compound acts through the same Xc system. This scenario is supported by our observation of a decrease in the concentration (compared with the control) of most metals, including iron, in the serum^36^. The question arises whether therapeutic interventions aimed at reducing elevated citric acid levels could mitigate neurodegenerative processes. In our studies, we observed the body’s response to a gene mutation that can lead to ferroptosis. However, a precise analysis of ferroptosis mechanisms requires an in vitro model developed from hiPSC-derived cells obtained from PEKAN and healthy patients.

## Conclusion

Our findings suggest that serum metabolomics could indicate ferroptosis and could be an essential cause of cell death in PKAN patients. Serum metabolomics also revealed different groups of PKAN patients with high citrate levels and low citrate levels, although the genetic mutations may be the same. We observed correlations between the clinical PKAN scale score and metabolites/metals that were significantly different between patients and controls.

## Materials and methods

### Patients

This study was observational. Twelve PKAN patients (all adult patients in Poland) and 12 controls participated in the study (4 males and 8 females per group). All PKAN patients received medical care from the 2nd Department of the Neurology Institute of Psychiatry and Neurology. In all patients, the disease was genetically confirmed. The demographic and clinical data are presented in Table 1. The mean age of symptom onset was 8 years. At the time of assessment, the mean age of the patients was 26 years. The first symptoms (gait disturbances) were noted in late childhood, except for two homozygotic patients who were born with hypotonia. The time interval from the first symptom to diagnosis was approximately 1.5–2 years. Clinical assessment was performed with the Disability Rating Scale (PKAN-DRS I-VI)^41^. The scale consists of 6 parameters assessing daily living activities and neurological symptoms. The assessed activities and symptoms are I—cognition, II—behavior, III—disability, IV—parkinsonism, V—dystonia, and VI—other symptoms such as speech, chorea, and spasticity. The patient could score points for each scale parameter, where 0 is no symptoms. Most patients had deep brain stimulation (DBS), but despite this, all had severe dystonia. The control group consisted of healthy volunteers (n = 12) whose age and sex were similar to those of the study group. All participants provided full, informed consent following a thorough explanation of all the examinations. The study was approved by the Ethical Committee of the Institute of Psychiatry and Neurology (Agreement 3/2022).

### NMR spectroscopy and metal level measurements

Serum samples were obtained from blood clots collected after overnight fasting and frozen at − 80 °C until NMR analysis was performed. Hydrophilic compounds were measured in the raw serum (600 μl) without any additional preparation. The pH of each sample was adjusted to 7.5 ± 0.2 with HCl. 3-Trimethylsilyl propionic acid (TSP) at a final concentration of 1 mM was used as an external reference for normalization of all the spectra and for quantitative statistical analysis. The hydrophobic compounds were prepared using a modified Bligh and Dyer method according to the procedure described in our previous publication^14^. A volume of 500 μl of serum was extracted for NMR spectrometry and vortexed for 5 min with 1875 μl of a mixture of 99% methanol, 98% chloroform, 36% HCl, and 40:20:1 (v/v). Next, 625 μl of chloroform was added, and the mixture was vortexed again for 5 min. Afterwards, 625 μl of water was added, and the mixture was vortexed for 5 min. Then, the mixture was centrifuged at 2000 × g for 30 min using a swing-out rotor to obtain three phases: upper, water/methanol containing amino acids and other substances diluted in water; lower, containing lipids; and middle, containing proteins. The lower phases were extracted for NMR examination. Before the levels of hydrophobic compounds were measured via an NMR spectrometer, the chloroform phase of each sample was dried with nitrogen. The dry residues were then diluted in 700 μl of CDCl_3_ and immediately tested. All chemicals used were purchased from Sigma, Germany.

All nuclear magnetic resonance (NMR) spectra were obtained at 25 °C using an Avance III HD 500 MHz (Bruker, Germany) spectrometer. We used a spin‒echo CPMG pulse sequence to collect proton NMR spectra for hydrophilic compounds and a one-pulse sequence for hydrophobic compounds. We measured hydrophilic compounds via 256 transients and a 12 s pulse repetition time, whereas chloroform extracts were measured via 128 transients and a 5 s pulse repetition time. Using vendor software (TopSpin v.3.6, Bruker, Germany), we applied a line broadening of 0.3, baseline, and phase correction to each spectrum. We also used 2D homonuclear COSY and heteronuclear 1H-31P HSQC spectra for signal assignment. NMR signals were assigned according to our own reference database, the Human Metabolome Database (HMDB), and the literature^14,42^. We expressed quantities of metabolites as magnitudes of signals corresponding to compound concentrations and normalized spectra to TSP or chloroform signals before statistical analysis. For the NMR data selection, we used a custom-written application (IBBE PAS). For the statistical analysis, we selected 39 compound signals from the raw serum samples and 30 functional group/compound signals from their lipid extracts.

Metal levels were measured according to the protocol described in our previous publication^42^. We diluted the serum samples with deionized water according to the measurement range of the analyzed elements (from 10- to 1000-fold depending on the element). We performed isotope-specific detection via mass spectrometry (ICP–MS) (Nexion 300D, Perkin Elmer, USA) using the parameters listed in the supplementary material (SM) Table 4 SM. We calculated the LOQ for each element on the basis of the formula recommended by the IUPAC^43^. We used an external calibration approach during ICP–MS quantification, and the solution was prepared by diluting the Merck VIII standard for ICP (Merck, Germany). For calculations, we took into account the mean elemental content from triplicate samples. We recalculated the results into molar concentrations.

### Statistical analysis

For all the data, we performed univariate statistical analysis. For the NMR data, one-way ANOVA followed by Holm‒Sidak or Dunn’s correction (depending on the Shapiro‒Wilk and equal variance test results) was used; for the metal ions, one-way ANOVA was performed for three groups, followed by Dunn’s correction. The same tests were performed for two-group analyses, also depending on the Shapiro‒Wilk test and equal variance test results. All univariate tests were performed via the Statistica ver. 10 package. P values lower than 0.05 were considered significant. Clinical parameters were correlated with biochemical and metal data via Spearman rank correlation (SM2). The chord diagram (Origin Pro 2023, OriginLab) presents statistically significant (*p* < 0.05) values of correlation coefficients, and Table 6 shows their directions (positive or negative correlation). We conducted a multivariate analysis (MVA) to further analyze the data. We employed an MVA projection method called orthogonal partial least squares-discriminant analysis (OPLS-DA), which is a classification technique based on the PLS regression algorithm. This method aims to maximize the covariance between the independent variables (X)—the original data—metabolites or metals—and the dependent variable (Y)—the groups or responses. This technique separates systematic X variation into two parts: one that is correlated to Y (predictive component) and reflects between-group variation and the other that is orthogonal or uncorrelated to Y (orthogonal component) and reflects within-group variation. This method helps us build a classification model and optimize separation between groups of subjects. It also provides a visual interpretation of such separation through a score plot, which is easily interpretable. An ellipse is drawn on the score plot to represent the Hotelling T-square test with 95% confidence in the model. Scores that lie significantly outside the Hotelling T-square ellipse are removed as outliers. The scores within the ellipse represent the observations on the plot that are close to each other and are more similar than the observations that are more distant from each other. The distance between scores in the vertical direction indicates within-group variation. We assessed the model performance using R^2^, which explains the total variation in the data and estimates the goodness of fit, and Q^2^, which is an internal cross-validation parameter that estimates the goodness of fit of the prediction. We considered the model to be positively validated when R^2^ and Q^2^ were equal to or greater than 0.5. The variable importance in the projection (VIP) value of each variable in the model was calculated to indicate its contribution to the classification. Variables with VIP values greater than 1.0 were considered to be significantly different^44^. Models were validated using CV-ANOVA tests via the jackknife method. We performed multivariate analysis via the software package SIMCA-P (ver. 15, Sartorius, Sweden).

## Electronic supplementary material

Below is the link to the electronic supplementary material.

Supplementary Material 1

Supplementary Material 2

## Data Availability

The data that support the findings of this study are available from the corresponding author upon reasonable request.

## References

[1] Gregory, A. & Hayflick, S. J. Genetics of neurodegeneration with brain iron accumulation. *Curr. Neurol. Neurosci. Rep.* **11**, 254–261. 10.1007/s11910-011-0181-3 (2011). 21286947 10.1007/s11910-011-0181-3 PMC5908240

[2] Schneider, S. A., Hardy, J. & Bhatia, K. P. Syndromes of neurodegeneration with brain iron accumulation (NBIA): An update on clinical presentations, histological and genetic underpinnings, and treatment considerations. *Mov. Disord.* **27**, 42–53. 10.1002/mds.23971 (2012). 22031173 10.1002/mds.23971

[3] Gregory, A., Polster, B. J. & Hayflick, S. J. Clinical and genetic delineation of neurodegeneration with brain iron accumulation. *J. Med. Genet.* **46**, 73–80. 10.1136/jmg.2008.061929 (2009). 18981035 10.1136/jmg.2008.061929 PMC2675558

[4] Schneider, S. A., Zorzi, G. & Nardocci, N. Pathophysiology and treatment of neurodegeneration with brain iron accumulation in the pediatric population. *Curr. Treat. Options Neurol.* **15**, 652–667. 10.1007/s11940-013-0254-5 (2013). 23888388 10.1007/s11940-013-0254-5

[5] Hayflick, S. J. et al. Genetic, clinical, and radiographic delineation of Hallervorden-Spatz syndrome. *N. Engl. J. Med.* **348**, 33–40. 10.1056/NEJMoa020817 (2003). 12510040 10.1056/NEJMoa020817

[6] Aggarwal, A. et al. Indian-subcontinent NBIA: Unusual phenotypes, novel PANK2 mutations, and undetermined genetic forms. *Mov. Disord.* **25**, 1424–1431. 10.1002/mds.23095 (2010). 20629144 10.1002/mds.23095

[7] Yoon, W. T., Lee, W. Y., Shin, H. Y., Lee, S. T. & Ki, C. S. Novel PANK2 gene mutations in Korean patient with pantothenate kinase-associated neurodegeneration presenting unilateral dystonic tremor. *Mov. Disord.* **25**, 245–247. 10.1002/mds.22891 (2010). 20014113 10.1002/mds.22891

[8] Chung, S. J., Lee, J. H., Lee, M. C., Yoo, H. W. & Kim, G. H. Focal hand dystonia in a patient with PANK2 mutation. *Mov. Disord.* **23**, 466–468. 10.1002/mds.21880 (2008). 18074375 10.1002/mds.21880

[9] Colombelli, C., Aoun, M. & Tiranti, V. Defective lipid metabolism in neurodegeneration with brain iron accumulation (NBIA) syndromes: Not only a matter of iron. *J. Inherit. Metab. Dis.* **38**, 123–136. 10.1007/s10545-014-9770-z (2015). 25300979 10.1007/s10545-014-9770-z

[10] KEEG. www.kegg.jp/kegg/kegg1.html

[11] Toczylowska, B., Zieminska, E., Polowy, R., Olszynski, K. H. & Lazarewicz, J. W. NMR-based metabolomics of rat hippocampus, serum, and urine in two models of autism. *Mol. Neurobiol.* **59**, 5452–5475. 10.1007/s12035-022-02912-5 (2022). 35715683 10.1007/s12035-022-02912-5

[12] Toczylowska, B., Zieminska, E., Senator, P. & Lazarewicz, J. W. Hippocampal metabolite profiles in two rat models of autism: NMR-based metabolomics studies. *Mol. Neurobiol.* **57**, 3089–3105. 10.1007/s12035-020-01935-0 (2020). 32468248 10.1007/s12035-020-01935-0 PMC7320041

[13] Zieminska, E., Ruszczynska, A., Augustyniak, J., Toczylowska, B. & Lazarewicz, J. W. Zinc and copper brain levels and expression of neurotransmitter receptors in two rat ASD models. *Front. Mol. Neurosci.* **14**, 656740. 10.3389/fnmol.2021.656740 (2021). 34267627 10.3389/fnmol.2021.656740 PMC8277171

[14] Toczylowska, B., Zieminska, E., Michalowska, M., Chalimoniuk, M. & Fiszer, U. Changes in the metabolic profiles of the serum and putamen in Parkinson’s disease patients—In vitro and in vivo NMR spectroscopy studies. *Brain Res.* **1748**, 147118. 10.1016/j.brainres.2020.147118 (2020). 32931820 10.1016/j.brainres.2020.147118

[15] Vanden Berghe, T., Linkermann, A., Jouan-Lanhouet, S., Walczak, H. & Vandenabeele, P. Regulated necrosis: The expanding network of non-apoptotic cell death pathways. *Nat. Rev. Mol. Cell Biol.* **15**, 135–147. 10.1038/nrm3737 (2014). 24452471 10.1038/nrm3737

[16] Feng, H. & Stockwell, B. R. Unsolved mysteries: How does lipid peroxidation cause ferroptosis?. *PLoS Biol.* **16**, e2006203. 10.1371/journal.pbio.2006203 (2018). 29795546 10.1371/journal.pbio.2006203 PMC5991413

[17] Lee, J. Y., Kim, W. K., Bae, K. H., Lee, S. C. & Lee, E. W. Lipid metabolism and ferroptosis. *Biology (Basel)*10.3390/biology10030184 (2021). 33801564 10.3390/biology10030184 PMC8000263

[18] Aoun, M. et al. Changes in red blood cell membrane lipid composition: A new perspective into the pathogenesis of PKAN. *Mol. Genet. Metab.* **121**, 180–189. 10.1016/j.ymgme.2017.04.006 (2017). 28456385 10.1016/j.ymgme.2017.04.006

[19] Cao, J. Y. & Dixon, S. J. Mechanisms of ferroptosis. *Cell Mol. Life Sci.* **73**, 2195–2209. 10.1007/s00018-016-2194-1 (2016). 27048822 10.1007/s00018-016-2194-1 PMC4887533

[20] Danek, A. et al. Neuroacanthocytosis: New developments in a neglected group of dementing disorders. *J. Neurol. Sci.* **229–230**, 171–186. 10.1016/j.jns.2004.11.024 (2005). 15760637 10.1016/j.jns.2004.11.024

[21] Chen, Z., Jiang, J., Fu, N. & Chen, L. Targetting ferroptosis for blood cell-related diseases. *J. Drug Target* **30**, 244–258. 10.1080/1061186X.2021.1971237 (2022). 34415804 10.1080/1061186X.2021.1971237

[22] Tarnacka, B., Jopowicz, A. & Maslinska, M. Copper, iron, and manganese toxicity in neuropsychiatric conditions. *Int. J. Mol. Sci.*10.3390/ijms22157820 (2021). 34360586 10.3390/ijms22157820 PMC8346158

[23] Santambrogio, P. et al. Massive iron accumulation in PKAN-derived neurons and astrocytes: Light on the human pathological phenotype. *Cell Death Dis.* **13**, 185. 10.1038/s41419-022-04626-x (2022). 35217637 10.1038/s41419-022-04626-x PMC8881507

[24] Clayton, P. T. Inherited disorders of transition metal metabolism: An update. *J. Inherit. Metab. Dis.* **40**, 519–529. 10.1007/s10545-017-0030-x (2017). 28303424 10.1007/s10545-017-0030-x

[25] Ferreira, C. R. & Gahl, W. A. Disorders of metal metabolism. *Transl. Sci. Rare Dis.* **2**, 101–139. 10.3233/TRD-170015 (2017). 29354481 10.3233/TRD-170015 PMC5764069

[26] Teschke, R. Aluminum, arsenic, beryllium, cadmium, chromium, cobalt, copper, iron, lead, mercury, molybdenum, nickel, platinum, thallium, titanium, vanadium, and zinc: Molecular aspects in experimental liver injury. *Int. J. Mol. Sci.*10.3390/ijms232012213 (2022). 36293069 10.3390/ijms232012213 PMC9602583

[27] Leoni, V. et al. Metabolic consequences of mitochondrial coenzyme A deficiency in patients with PANK2 mutations. *Mol. Genet. Metab.* **105**, 463–471. 10.1016/j.ymgme.2011.12.005 (2012). 22221393 10.1016/j.ymgme.2011.12.005 PMC3487396

[28] Tuschl, K., Mills, P. B. & Clayton, P. T. Manganese and the brain. *Int. Rev. Neurobiol.* **110**, 277–312. 10.1016/B978-0-12-410502-7.00013-2 (2013). 24209443 10.1016/B978-0-12-410502-7.00013-2

[29] Vellingiri, B. et al. Influence of heavy metals in Parkinson’s disease: An overview. *J. Neurol.* **269**, 5798–5811. 10.1007/s00415-022-11282-w (2022). 35900586 10.1007/s00415-022-11282-w

[30] Yildirim, C. et al. Investigation covering the effect of boron plus taurine application on protein carbonyl and advanced oxidation protein products levels in experimental Alzheimer model. *Biol. Trace Elem. Res.* **201**, 1905–1912. 10.1007/s12011-022-03293-5 (2023). 35618890 10.1007/s12011-022-03293-5

[31] Farrin, N. et al. Boron compound administration; A novel agent in weight management: A systematic review and meta-analysis of animal studies. *J. Trace Elem. Med. Biol.* **72**, 126969. 10.1016/j.jtemb.2022.126969 (2022). 35298949 10.1016/j.jtemb.2022.126969

[32] Costello, L. C. & Franklin, R. B. Plasma citrate homeostasis: how it is regulated; and its physiological and clinical implications. An important, but neglected, relationship in medicine. *HSOA J. Hum. Endocrinol.* **1**, 005 (2016). 28286881 PMC5345696

[33] Fan, S. Z. et al. Dietary citrate supplementation enhances longevity, metabolic health, and memory performance through promoting ketogenesis. *Aging Cell* **20**, e13510. 10.1111/acel.13510 (2021). 34719871 10.1111/acel.13510 PMC8672782

[34] Werning, M. et al. A potential citrate shunt in erythrocytes of pkan patients caused by mutations in pantothenate kinase 2. *Biomolecules*10.3390/biom12020325 (2022). 35204826 10.3390/biom12020325 PMC8869601

[35] Stephenson, E., Nathoo, N., Mahjoub, Y., Dunn, J. F. & Yong, V. W. Iron in multiple sclerosis: Roles in neurodegeneration and repair. *Nat. Rev. Neurol.* **10**, 459–468. 10.1038/nrneurol.2014.118 (2014). 25002107 10.1038/nrneurol.2014.118

[36] Wang, H. et al. Characterization of ferroptosis in murine models of hemochromatosis. *Hepatology* **66**, 449–465. 10.1002/hep.29117 (2017). 28195347 10.1002/hep.29117 PMC5573904

[37] Baba, Y. et al. Protective effects of the mechanistic target of rapamycin against excess iron and ferroptosis in cardiomyocytes. *Am. J. Physiol. Heart Circ. Physiol.* **314**, H659–H668. 10.1152/ajpheart.00452.2017 (2018). 29127238 10.1152/ajpheart.00452.2017 PMC5899260

[38] Dezfouli, M. A. et al. Pantothenate kinase 2 mutation with eye-of-the-tiger sign on magnetic resonance imaging in three siblings. *Iran. J. Neurol.* **11**, 155–158 (2012). 24250886 PMC3829266

[39] Thomas, G. E. C. et al. Regional brain iron and gene expression provide insights into neurodegeneration in Parkinson’s disease. *Brain* **144**, 1787–1798. 10.1093/brain/awab084 (2021). 33704443 10.1093/brain/awab084 PMC8320305

[40] Nagasawa, K. et al. Transport mechanism for aluminum citrate at the blood-brain barrier: Kinetic evidence implies involvement of system Xc-in immortalized rat brain endothelial cells. *Toxicol. Lett.* **155**, 289–296. 10.1016/j.toxlet.2004.10.004 (2005). 15603924 10.1016/j.toxlet.2004.10.004

[41] Darling, A. et al. Clinical rating scale for pantothenate kinase-associated neurodegeneration: A pilot study. *Mov. Disord.* **32**, 1620–1630. 10.1002/mds.27129 (2017). 28845923 10.1002/mds.27129

[42] Toczylowska, B., Zieminska, E., Podlecka-Pietowska, A., Ruszczynska, A. & Chalimoniuk, M. Serum metabolic profiles and metal levels of patients with multiple sclerosis and patients with neuromyelitis optica spectrum disorders—NMR spectroscopy and ICP-MS studies. *Mult. Scler. Relat. Disord.* **60**, 103672. 10.1016/j.msard.2022.103672 (2022). 35240533 10.1016/j.msard.2022.103672

[43] Currie, L. A. Nomenclature in evaluation of analytical methods including detection and quantification capabilities (Iupac recommendations 1995). *Pure Appl. Chem.* **67**, 1699–1723. 10.1351/pac199567101699 (1995).

[44] Toczylowska, B., Jamrozik, Z., Liebert, A. & Kwiecinski, H. NMR-based metabonomics of cerebrospinal fluid applied to amyotrophic lateral sclerosis. *Biocybern. Biomed. Eng.* **33**, 21–32. 10.1016/S0208-5216(13)70053-6 (2013).

