# Supplementary material for: Serum metabolomics indicates ferroptosis in patients with pantothenate kinase associated neurodegeneration
Source: Sci Rep. 2025 Mar 20;15:9592. doi: 10.1038/s41598-025-94838-w (PMC11926261; doi:10.1038/s41598-025-94838-w)
Supplement: Supplementary file 1 — Supplementary Material 1 [file 41598_2025_94838_MOESM1_ESM.docx]

Serum metabolomics indicates ferroptosis in patients with pantothenate kinase-associated neurodegeneration

Beata Toczylowska^1^, Marta Skowronska^2^, Iwona Kurkowska-Jastrzebska^2^, Anna Ruszczynska^3^, Elzbieta Zieminska ^4*^

1. Nalecz Institute of Biocybernetics and Biomedical Engineering, PAS, Ks. Trojdena 4 st., 02-109 Warsaw, Poland; [beata.toczylowska@ibib.waw.pl](mailto:beata.toczylowska@ibib.waw.pl)
2. 2^nd^ Department of Neurology, Institute of Psychiatry and Neurology, Sobieskiego 9 st., 02-957 Warsaw, Poland; [mskowronska@ipin.edu.pl](mailto:mskowronska@ipin.edu.pl); [ikurkowska@ipin.edu.pl](mailto:ikurkowska@ipin.edu.pl)
3. University of Warsaw, Faculty of Chemistry, Biological and Chemical Research Centre, Zwirki i Wigury 101 st., 02-089 Warsaw, Poland; [aruszcz@chem.uw.edu.pl](mailto:aruszcz@chem.uw.edu.pl)
4. Mossakowski Medical Research Institute, PAS, A. Pawinskiego 5 st., 02-106 Warsaw, Poland; [elziem@imdik.pan.pl](mailto:elziem@imdik.pan.pl)

*Corresponding author: Elzbieta Zieminska, Mossakowski Medical Research Institute, PAS, 02-106 Warsaw, Poland, tel. +48 22 608 65 33; [elziem@imdik.pan.pl](mailto:elziem@imdik.pan.pl) ; orcid: 0000-0002-5817-5650

Supplementary material

Table 1 SM. Statistical analyses of differences between the PKAN subgroups of hydrophilic compounds. The data are presented as mean±SD or median(25%-75%) – depending on the test used of the PKAN patient subgroup vs control levels and between PKAN subgroups; for each analysis ANOVA test p values are presented with bold indicated significant differences. P1 – n=3, P2 – n=2, P3 – n=7, control n=12. Not tested – no possible measurement of signal intensity in P1 group.

| compound | P3 vs control [a.u.] | P | P1 vs control  [a.u.] | P | P2 vs control  [a.u.] | P | P1 vs P3  [a.u.] | P | P2 vs P3  [a.u.] | P |
| --- | --- | --- | --- | --- | --- | --- | --- | --- | --- | --- |
| Formate | 1869.0(1661.0-3458.0)/  519.5(375.25-1202.0) | **0.001** | 1752.0(1732.0-1818.0)/  519.5(375.25-1202.0) | **0.009** | 1953.0(1862.0-2044.0)/  519.5(375.25-1202.0) | **0.028** | 1767.333±45.004/  2303.571±1051.316 | 0.418 | 1953.000±128.693/  2303.571±1051.316 | 0.667 |
| Histidine | 1787.0(1596.0-1897.0)/  2555.5(2145.75-2948.25) | **0.014** | 1446.0(1197.0-1678.0)/  2555.5(2145.75-2948.25) | **0.030** | 2607.167±1055.239/  2425.750±807.624 | 0.780 | 1440.333±240.55/  1725.571±553.176 | 0.427 | 2607.167±1055.239/  1725.571±553.176 | 0.134 |
| Phenylalanine | 548.0(492.0-602.0)/  410.5(347.75-510.0) | 0.052 | 529.667±9.746/  445.417±143.197 | 0.356 | 643.000±49.497/  445.417±143.197 | 0.085 | 529.667±9.746/  535.714±63.226 | 0.905 | 643.000±49.497/  535.714±63.226 | 0.066 |
| Tyrosine | 1144.857±219.534/  987.167±180.755 | 0.108 | 1315.0±246.639/  987.167±180.755 | **0.020** | 1761.167±22.863/  987.167±180.755 | **0.001** | 1315.0±246.639/  1144.857±219.534 | 0.308 | 1761.167±22.863/  1144.857±219.534 | **0.007** |
| Urea | 9061.143±1923.983/  8857.167±4605.857 | 0.913 | 10738.667±2899.079/  8857.167±4605.857 | 0.518 | 7090.0±6065.562/  8857.167±4605.857 | 0.635 | 10738.667±2899.079/  9061.143±1923.983 | 0.303 | 7090.0±6065.562/  9061.143±1923.983 | 0.425 |
| Cis-aconitate | 291.0(222.0-368.0)/  0.0(0.0-0.0) | **0.001** | 0.0(0.0-0.0)/  0.0(0.0-0.0) | **1.00** | 642.667(552.0-733.333)/  0.0(0.0-0.0) | **0.001** | 0.0(0.0-0.0)/  291.0(222.0-368.0) | **0.033** | 642.667±128.222/  321.714±228.805 | 0.108 |
| α-D glucose | 28360.000(27241.0-28684.0)/  30182.5(27487.0-38969.5) | 0.272 | 27303.333±5413.62/  32079.583±7103.194 | 0.301 | 38729.167±8666.536/  32079.583±7103.194 | 0.253 | 27303.333±5413.62/  28388.143±1648.695 | 0.621 | 38729.167(32601.0-44857.333)/  28360.000(27241.0-28684.0) | 0.056 |
| Unassigned 4.03ppm | 1812.286±217.004/  2390.833±652.439 | **0.038** | 1794.333±368.392/  2390.833±652.439 | 0.158 | 2006.167±440.056/  2390.833±652.439 | 0.445 | 1794.333±368.39/  1812.286±217.004 | 0.924 | 2006.167±440.056/  1812.286±217.004 | 0.385 |
| X1 | 0.0(0.0-0.0)/  0.0(0.0-0.0) | 1.00 | 748211.0(175726.0-1201618.0)/  0.0(0.0-0.0) | **0.001** | 0.0(0.0-0.0)/  0.0(0.0-0.0) | 1.00 | 748211.0(175726.0-1201618.0)/  0.0(0.0-0.0) | **0.017** | 0.0(0.0-0.0)/  0.0(0.0-0.0) | 1.00 |
| Myo-inositol | 3103.429±634.373/  4375.25±874.265 | **0.004** | Not tested |  | 4635.167±477.768/  4375.25±874.265 | 0.695 | Not tested |  | 4635.167±477.768/  3103.429±634.373 | **0.017** |
| Glycine | 14035.0(13675.0-15906.0)/  33297.0(26901.25-36688.25) | **0.001** | 16754.0(13913.0-21673.0)/  33297.0(26901.25-36688.25) | **0.014** | 22438.833(8519.0-36358.667)/  33297.0(26901.25-36688.25) | 0.584 | 17446.667±3926.097/  15406.571±3350.211 | 0.423 | 22438.833(8519.0-36358.667)/  14035.0(13675.0-15906.0) | 1.00 |
| D-Glucose | 33843.571±2884.44/  40356.5±8670.686 | 0.074 | 36635.333±4448.909/  40356.5±8670.686 | 0.493 | 43666.333±7467.519/  40356.5±8670.686 | 0.623 | 36635.333±4448.909/  33843.571±2884.44 | 0.261 | 43666.333±7467.519/  33843.571±2884.44 | **0.016** |
| Glycerophosphocholine/Choline | 40843.0(37556.0-48801.0)/  56697.0(32271.0-75935.0) | 0.353 | 320577.0(157779.0-494785.0)/  56697.0(32271.0-75935.0) | **0.009** | 46568.0±17886.973/  57769.417±27984.156 | 0.601 | 320577.0(157779.0-494785.0)/  40843.0(37556.0-48801.0)/ | **0.017** | 46568.0(33920.0-59216.0)/  40843.0(37556.0-48801.0)/ | 1.00 |
| 9-methyluric acid | 3222.286±1367.775/  2351.25±1029.35 | 0.133 | Not tested | - | 1789.667±1289.291/  2351.25±1029.35 | 0.498 | Not tested | - | 1789.667±1289.291/  3222.286±1367.775 | 0.229 |
| Malonic acid | 2861.0(2545.0-3244.0)/  595.0(463.75-714.0) | **0.001** | Not tested | - | 3410.667(3152.0-3669.333)/  595.0(463.75-714.0) | **0.028** | Not tested | - | 3410.667±365.810/  2838.857±382.657 | 0.103 |
| X2 | 0.0(0.0-0.0)/  0.0(0.0-0.0) | 1.00 | 101633.0(99995.0-106635.0)/  0.0(0.0-0.0) | **0.001** | 0.0(0.0-0.0)/  0.0(0.0-0.0) | 1.00 | 101633.0(99995.0-106635.0)/  0.0(0.0-0.0) | **0.017** | 0.0(0.0-0.0)/  0.0(0.0-0.0) | 1.00 |
| L-cysteine | 497.0(394.0-1196.0)/  532.50(92.5-702.25) | 0.800 | Not tested | - | 1050.333±288.971/  544.250±173.862 | **0.004** | Not tested | - | 1050.333(846.0-1254.667)/  497.0(394.0-1196.0) | 0.333 |
| Creatine/Creatinine | 6176.0(4343.0-7885.0)/  7093.0(5736.25-7896.5) | 0.310 | 10325.333±1893.163/  6883.75±1109.102 | **0.001** | 11393.833(6778.667-16009.0)/  7093.0(5736.25-7896.5) | 0.361 | 10325.333±1893.163/  6330.714±2161.728 | **0.025** | 11393.833(6778.667-16009.0)/  6176.0(4343.0-7885.0) | 0.333 |
| X3 | 0.0(0.0-0.0)/  0.0(0.0-0.0) | 1.00 | 55460.0(53449.0-62497.0)/  0.0(0.0-0.0) | **0.001** | 0.0(0.0-0.0)/  0.0(0.0-0.0) | 1.00 | 55460.0(53449.0-62497.0)/  0.0(0.0-0.0) | **0.017** | 0.0(0.0-0.0)/  0.0(0.0-0.0) | 1.00 |
| Citrate | 365175.0(313494.0-402971.0)/  2413.5(1413.0-2785.75) | **0.001** | 138587.0(3050.0-147672.0)/  2413.5(1413.0-2785.75) | **0.021** | 659631.5(604291.0-714972.0)/  2413.5(1413.0-2785.75) | **0.028** | 6436.3339±81002.406/  360011.0±51165.177 | **0.0002** | 659631.5±78263.286/  360011.0±51165.177 | **0.0003** |
| X4 | 0.0(0.0-0.0)/  0.0(0.0-0.0) | 1.00 | 122616.0(122541.0-129250.0)/  0.0(0.0-0.0) | **0.001** | 0.0(0.0-0.0)/  0.0(0.0-0.0) | 1.00 | 122616.0(122541.0-129250.0)/  0.0(0.0-0.0) | **0.017** | 0.0(0.0-0.0)/  0.0(0.0-0.0) | 1.00 |
| L-Glutamine | 5780.857±1299.493/  6652.083±3030.024 | 0.483 | 6115.333±999.849/  6652.083±3030.024 | 0.772 | 4350.5±2219.608/  6652.083±3030.024 | 0.330 | 6115.333±999.84/  5780.857±1299.493 | 0.704 | 4350.5±2219.608/  5780.857±1299.493 | 0.263 |
| Pyruvate | 3568.714±1182.924/  3223.5±1043.463 | 0.591 | 2256.667±678.923/  3223.5±1043.463 | 0.195 | 5647.667(3037.333-8258.0)/  3544.0(2088.-4030.25) | 0.643 | 2256.667±678.92/  3568.714±1182.924 | 0.116 | 5647.667(3037.333-8258.0)/  3360.0(2992.0-4470.0) | 0.667 |
| L-Glutamate | 1108.0(886.0-1433.0)/  2615.5(2192.0-4920.75) | **0.001** | 914.0(895.0-1334.0)/  2615.5(2192.0-4920.75) | **0.009** | 3044.667(1985.333-4104.0)/  2615.5(2192.0-4920.75) | 0.584 | 3568.714±1182.924/  1247.0±465.242 | 0.513 | 3044.667(1985.333-4104.0)/  1108.0(886.0-1433.0) | 0.111 |
| Acetone | 11515.0(9320.0-23618.0)/  5670.5(4578.75-8036.5) | **0.011** | 11629.000±3131.625/  6586.25±3759.554 | 0.053 | 19217.667(11985.333-26450.0)/  5670.5(4578.75-8036.5) | **0.045** | 12813.0(8078.0-13996.0)/  11515.0(9320.0-23618.0) | 1.00 | 19217.667(11985.333-26450.0)/  11515.0(9320.0-23618.0) | 0.333 |
| Methionine | 6192.286±950.865/  7365.917±3061.081 | 0.342 | 6541.333±832.236/  7365.917±3061.081 | 0.660 | 7452.333±4025.323/  7365.917±3061.081 | 0.972 | 6541.333±832.23/  6192.286±950.865 | 0.598 | 7452.333(4606.0-10298.667)/  6057.0(5438.0-6750.0) | 1.00 |
| N-Acetyl glycoprotein | 29090.429±2005.573/  36183.583±7342.933 | **0.024** | 32135.333±8517.534/  36183.583±7342.933 | 0.420 | 31416.0±859.842/  36183.583±7342.933 | 0.392 | 28962.0(25660.0-41784.0)/  29353.0(27174.0-30944.0) | 1.00 | 31416.0(30808.0-32024.0)/  29353.0(27174.0-30944.0) | 0.222 |
| Acetate | 2581.0(1578.0-4113.0)/  3009.0(1517.75-6429.5) | 0.673 | 4110.667±1187.391/  3588.417±2632.869 | 0.748 | 3052.5(2453.0-3652.0)/  3009.0(1517.75-6429.5) | 0.855 | 4110.667±1187.391/  3050.714±1818.803 | 0.388 | 3052.500±847.821/  3050.714±1818.803 | 0.999 |
| GABA | 432.0(416.0-617.0)/  1003.0(913.25-1221.75) | **0.005** | 767.0±255.106/  1044.5±274.839 | 0.138 | 1135.167±81.789/  1044.5±274.839 | 0.661 | 767.0±255.106/  571.143±273.186 | 0.322 | 1135.167(1077.333-1193.0)/  432.0(416.0-617.0) | 0.111 |
| Lysine | 1052.0(908.0-1291.0)/  1421.0(1296.25-1900.75) | **0.018** | 1297.333±17.616/  1585.333±368.033 | 0.210 | 2234.5±552.2/  1585.333±368.033 | **0.048** | 1292.0(1283.0-1317.0)/  1052.0(908.0-1291.0) | 0.183 | 2234.5(1844.0-2625.0)/  1052.0(908.0-1291.0) | 0.111 |
| Lipids (VLDL) | 825.0(562.0-2111.0)/  1968.0(846.25-2777.25) | 0.236 | 1378.0(0.0-1465.0)/  1968.0(846.25-2777.25) | 0.111 | 773.50(0.0-1547.0)/  1968.0(846.25-2777.25) | 0.142 | 947.667±821.855/  1155.286±1011.561 | 0.764 | 773.5±1093.894/  1155.286±1011.561 | 0.656 |
| Alanine | 16216.857±6655.530/  24099.5±4135.716 | **0.005** | 12246.667±3729.036/  24099.5±4135.716 | **0.001** | 21996.333±4811.626/  24099.5±4135.716 | 0.524 | 12246.667±3729.036/  16216.857±6655.530 | 0.370 | 21996.333±4811.626/  16216.857±6655.530 | 0.299 |
| Lactate | 46614.0(24927.0-75450.0)/  51794.0(42175.25-71365.0) | 0.499 | 43573.333±15497.320/  56181.167±16287.477 | 0.249 | 100228.667±10237.963/  56181.167±16287.477 | **0.003** | 43573.333±15497.320/  55915.571±36355.449 | 0.596 | 100228.667±10237.963/  55915.571±36355.449 | 0.147 |
| Lipids CH_2_ (LDL and VLDL) | 43506.0(33705.0-73052.0)/  76919.5(33136.25-96782.5) | 0.398 | 56233.0(28457.0-70180.0)/  76919.5(33136.25-96782.5) | 0.248 | 50481.167(42033.333-58929.0)/  76919.5(33136.25-96782.5) | 0.465 | 51623.333±21240.032/  51477.714±22546.466 | 0.993 | 50481.167±11947.04/  51477.714±22546.466 | 0.955 |
| 3-OH butyrate | 8784.0(588.0-12648.0)/  535.5(343.0-13882.5) | 0.673 | 447.0(0.0-9768.0)/  535.5(343.0-13882.5) | 0.312 | 2640.667(404.0-4877.333)/  535.5(343.0-13882.5) | 0.855 | 3405.0±5515.05/  7396.143±7020.965 | 0.412 | 2640.667±3163.124/  7396.143±7020.965 | 0.399 |
| Valine | 7563.571±909.084/  10068.833±1853.672 | **0.004** | 7765.667±2108.27/  10068.833±1853.672 | 0.082 | 10812.333±2412.177/  10068.833±1853.672 | 0.619 | 7765.667±2108.2/  7563.571±909.084 | 0.829 | 10812.333±2412.177/  7563.571±909.084 | **0.014** |
| Isoleucine | 1404.0(1387.0-1823.0)/  1734.0(1520.5-2345.25) | 0.237 | 1423.0(1256.0-2495.0)/  1734.0(1520.5-2345.25) | 0.386 | 2786.0±1388.758/  1889.250±551.587 | 0.102 | 1423.0(1256.0-2495.0)/  1404.0(1387.0-1823.0) | 1.00 | 2786.0±1388.758/  1637.857±410.587 | 0.063 |
| Leucine | 2706.0(2498.0-3000.0)/  2604.5(2354.0-3728.0) | 0.735 | 2247.0(2093.0-3646.0)/  2604.5(2354.0-3728.0) | 0.312 | 4634.333±2035.996/  2979.250±950.816 | 0.069 | 2662.0±855.641/  2768.571±338.179 | 0.773 | 4634.333(3194.667-6074.0)/  2706.0(2498.0-3000.0) | 0.111 |
| Lipids CH_3_ (LDL,VLDL,HDL) | 33993.0(30349.0-40940.0)/  52325.0(24456.5-76755.0) | 0.205 | 40249.0±7392.926/  50649.75±26775.466 | -0.527 | 36435.667±4493.899/  50649.75±26775.466 | 0.482 | 40249.0±7392.92/  36292.857±6993.385 | 0.442 | 36435.667±4493.899/  36292.857±6993.385 | 0.980 |

Table 2 SM. Statistical analyses of differences between the hydrophobic compound/functional group levels of PKAN subgroups. The data are presented as mean±SD or median(25%-75%) – depending on the test used of the PKAN patient subgroup vs control levels and between PKAN subgroups; for each analysis ANOVA test p values are presented with bold indicated significant differences. P1 – n=3, P2 – n=2, P3 – n=7, control n=12.

| Compound | P3 vs control |  | P1 vs control |  | P2 vs control |  | P1 vs P3 |  | P2 vs P3 |  |
| --- | --- | --- | --- | --- | --- | --- | --- | --- | --- | --- |
| Estriol | 236.782±106.369/  389.452±179.907 | 0.058 | 273.405±92.894/  389.452±179.907 | 0.308 | 188.988±136.573/  389.452±179.907 | 0.163 | 273.405±92.894/  236.782±106.369 | 0.621 | 188.988(92.416-285.559)/  293.0(120.9-328.0) | 0.333 |
| Estrone | 278.611±135.303/  236.701±186.832 | 0.612 | 307.73±141.67/  236.701±186.832 | 0.553 | 218.432±198.162/  236.701±186.832 | 0.901 | 373.0(145.19-405.0)/  304.0(136.259-400.0) | 0.833 | 218.432(78.31-358.554)/  304.0(136.259-400.0) | 0.50 |
| Testosterone | 97.0(84.349-283.0)/  124.07(59.623-211.0) | 0.499 | 232.0(125.45-452.0)/  124.07(59.623-211.0) | 0.149 | 102.507±60.358/  124.07(59.623-211.0) | 0.615 | 269.817±166.527/  178.352±115.702 | 0.339 | 102.507±60.358/  178.352±115.702 | 0.416 |
| Phosphatidylcholine Phosphatidylethanolamine Sphingomyelin  PUFA and MUFA | 39328.686±23106.517/  41465.251±31150.534 | 0.877 | 46912.315±16169.084/  41465.251±31150.534 | 0.778 | 25519.437±22110.969/  41465.251±31150.534 | 0.507 | 46912.315±16169.084/  39328.686±23106.517 | 0.624 | 25519.437±22110.969/  39328.686±23106.517 | 0.478 |
| 1,2-DAG, 2-MG | 810.051±461.745/  681.901±343.094 | 0.498 | 525.925±118.179/  681.901±343.094 | 0.462 | 642.756±368.326/  681.901±343.094 | 0.884 | 525.925±118.179/  810.051±461.745 | 0.338 | 642.756±368.326/  810.051±461.745 | 0.657 |
| Palmitic acid in FA | 5721.248±3567.462/  6656.076±4514.238 | 0.646 | 6721.961±2452.504/  6656.076±4514.238 | 0.981 | 3408.627±1791.820/  6656.076±4514.238 | 0.348 | 6721.961±2452.504/  5721.248±3567.462 | 0.674 | 3408.627±1791.82/  5721.248±3567.462 | 0.421 |
| 1,2-DAG | 3037.0(1797.24-6234.0)/  3401.903(1326.718-6461.75) | 0.933 | 5544.0(2777.085-5709.0)/  3401.903(1326.718-6461.75) | 0.773 | 2168.998(935.347-3402.65)/  3401.903(1326.718-6461.75) | 0.361 | 4676.695±1647.178/  4347.072±2720.821 | 0.853 | 2168.998±1744.64/  4347.072±2720.821 | 0.332 |
| Triglycerides | 4915.116(2178.997-7951.0)/  3714.561(2808.943-6287.75 | 0.80 | 5136.0(3636.765-10054.0)/  3714.561(2808.943-6287.75 | 0.248 | 2722.249(832.23-4612.267)/  3714.561(2808.943-6287.75 | 0.465 | 6275.588±3356.966/  5255.788±3435.533 | 0.677 | 2722.249±2672.88/  5255.788±3435.533 | 0.375 |
| 1,3-DAG | 4894.0(1894.252-5574.0)/  3521.119(0.0-5595.953) | 0.443 | 4663.0(2707.674-5630.0)/  3521.119(0.0-5595.953) | 0.462 | 3035.302(1575.45-4495.155)/  3521.119(0.0-5595.953) | 0.852 | 4663.0(2707.674-5630.0)/  4894.0(1894.252-5574.0) | 0.833 | 3035.302(1575.45-4495.155)/  4894.0(1894.252-5574.0) | 0.333 |
| 1-MAG | 4659.0(1897.521-5537.0)/  3152.307(0.0-5282.091) | 0.348 | 4712.0(2707.674-5530.0)/  3152.307(0.0-5282.091) | 0.304 | 2926.157(1412.506-4439.808)/  3152.307(0.0-5282.091) | 0.852 | 4712.0(2707.674-5530.0)/  4659.0(1897.521-5537.0) | 0.833 | 2926.157(1412.506-4439.808)/  4659.0(1897.521-5537.0) | 0.333 |
| Phosphatidylcholine | 51926.167(36433.079-127213.0)/  41284.5(16771.002-106831.5) | 0.398 | 63752.0(39334.499-76950.0)/  41284.5(16771.002-106831.5) | 0.773 | 37964.079(12282.086-63646.071)/  41284.5(16771.002-106831.5) | 0.584 | 60012.166±19084.581/  79493.177±49555.202 | 0.539 | 37964.079±36319.822/  79493.177±49555.202 | 0.315 |
| Sphingomyelin | 25851.218±16045.317/  30563.629±23947.893 | 0.658 | 30460.324±9760.77/  30563.629±23947.893 | 0.994 | 12965.146±11561.307/  30563.629±23947.893 | 0.347 | 30460.324±9760.77/  25851.218±16045.317 |  | 12965.146±11561.307/  25851.218±16045.317 | 0.334 |
| Phosphatidylethanolamine | 1783.0(857.801-1868.0)/  0.0(0.0-86.69) | **0.001** | 699.0(78.326-703.0)/  0.0(0.0-86.69 | **0.048** | 1500.681(314.214-687.147)/  0.0(0.0-86.69) | **0.015** | 493.442±359.506/  1609.759±757.398 | **0.045** | 1500.681±1677.91/  1609.759±757.398 | 0.890 |
| PUFAs | 11178.0(4946.151-18196.0)/  8200.223(3475.186-18009.5) | 0.499 | 14250.499±3900.324/  10891.748±9140.006 | 0.553 | 8508.318±7352.975/  10891.748±9140.006 | 0.735 | 14250.499±3900.324/  12246.224±7532.068 | 0.681 | 8508.318±7352.97/  12246.224±7532.068 | 0.554 |
| Linoleic acid | 15266.0(8926.99-28438.0)/  18952.162(8184.33-39436.0) | 0.933 | 24850.534±10728.34/  24340.753±19004.11 | 0.966 | 10417.783(3809.485-17026.082)/  18952.162(8184.33-39436.0) | 0.201 | 24850.534±10728.34/  20129.298±11069.504 | 0.551 | 10417.783±9345.546/  20129.298±11069.504 | 0.301 |
| Palmitic acid | 39939.170±26521.751/  39713.341±28948.324 | 0.987 | 44157.307±16600.939/  39713.341±28948.324 | 0.806 | 19497.685±14644.621/  39713.341±28948.324 | 0.364 | 44157.307±16600.939/  39939.170±26521.751 | 0.809 | 19497.685±14644.621/  39939.170±26521.751 | 0.345 |
| Hexanoylglycine | 4653.569(4244.733-13040.0)/  6010.304(2714.705-11165.75) | 0.447 | 9644.155±4096.987/  6706.639±4083.305 | 0.285 | 7686.724(2933.965-12439.484)/  6010.304(2714.705-11165.75) | 0.715 | 9644.155±4096.987/  7463.864±5368.804 | 0.551 | 7686.724±6721.41/  7463.864±5368.804 | 0.962 |
| FAs | 30527.146±16700.805/  33115.222±30508.415 | 0.840 | 38195.575±16431.332/  33115.222±30508.415 | 0.789 | 15244.058±13089.255/  33115.222±30508.415 | 0.442 | 37574.117±13504.866/  33857.804±18821.411 | 0.768 | 15244.058±13089.255/  30527.146±16700.805 | 0.279 |
| Vaccenic acid | 5418.739±3512.612/  6017.577±4312.12 | 0.760 | 6229.539±2158.337/  6017.577±4312.12 | 0.937 | 2885.653±2125.306/  6017.577±4312.12 | 0.345 | 220392.844±83854.658/  190995.781±107950.978 | 0.689 | 2885.653±2125.30/  5418.739±3512.612 | 0.377 |
| Palmitoleic acid | 4358.630±2141.367/  3456.877±2132.503 | 0.387 | 3546.582±790.043/  3456.877±2132.503 | 0.945 | 3199.505±2381.370/  3456.877±2132.503 | 0.878 | 699675.093±240314.923/  638575.559±360827.405 | 0.798 | 3199.505±2381.37/  4358.630±2141.367 | 0.528 |
| 1,3-DAG, 1-MAG | 33857.804±18821.411/  36853.667±25556.377 | 0.791 | 37574.117±13504.866/  36853.667±25556.377 | 0.964 | 19710.340±14727.305/  36853.667±25556.377 | 0.384 | 124198.983±43021.593/  107766.346±68203.224 | 0.715 | 19710.340±14727.305/  33857.804±18821.411 | 0.367 |
| Saturated FAs, PUFAs and MUFAs | 190995.781±107950.978/  203606.522±136950.476 | 0.838 | 220392.844±83854.658/  203606.522±136950.476 | 0.845 | 109621.973±87646.171/  203606.522±136950.476 | 0.375 | 220392.844±83854.658/  190995.781±107950.978 | 0.689 | 109621.973±87646.171/  190995.781±107950.978 | 0.367 |
| Saturated FAs and PUFAs | 638575.559±360827.405/  635208.583±428559.252 | 0.986 | 699675.093±240314.923/  635208.583±428559.252 | 0.809 | 358554.522±262539.149/  635208.583±428559.252 | 0.402 | 699675.093±240314.923/  638575.559±360827.405 | 0.798 | 358554.522±262539.149/  638575.559±360827.405 | 0.350 |
| Cholesterol esters | 107766.346±68203.224/  123857.748±81462.58 | 0.666 | 124198.983±43021.593/  123857.748±81462.58 | 0.995 | 57890.654±43857.86/  123857.748±81462.58 | 0.296 | 124198.983±43021.593/  107766.346±68203.224 | 0.715 | 57890.654±43857.86/  107766.346±68203.224 | 0.372 |
| Free cholesterol | 53311.128±34673.88/  56551.223±38554.376 | 0.857 | 61199.159±21798.469/  56551.223±38554.376 | 0.847 | 28526.451±21390.892/  56551.223±38554.376 | 0.346 | 61199.159±21798.469/  53311.128±34673.88 | 0.730 | 28526.451±21390.892/  53311.128±34673.88 | 0.382 |
| Saturated FAs, PUFAs and MUFAs | 171966.244±98812.014/  176283.381±112082.496 | 0.934 | 185898.582±61716.855/  176283.381±112082.496 | 0.890 | 98884.945±75175.381/  176283.381±112082.496 | 0.373 | 185898.582±61716.855/  171966.244±98812.014 | 0.830 | 98884.945±75175.381/  171966.244±98812.014 | 0.373 |
| Saturated FAs | 31152.604±15491.725/  30528.094±18776.59 | 0.942 | 27675.556±7429.686/  30528.094±18776.59 | 0.805 | 25214.618±17457.722/  30528.094±18776.59 | 0.716 | 27675.556±7429.686/  31152.604±15491.725 | 0.727 | 25214.618±17457.722/  31152.604±15491.725 | 0.653 |
| 24S–Hydroxycholesterol | 550.583±250.147/  560.214±306.391 | 0.945 | 743.023±472.153/  560.214±306.391 | 0.416 | 380.326±312.928/  560.214±306.391 | 0.458 | 743.023±472.153/  550.583±250.147 |  | 380.326±312.928/  550.583±250.147 | 0.441 |
| Free cholesterol and cholesterol esters | 134345.857±86667.744/  152139.821±102243.407 | 0.705 | 156686.992±53763.052/  152139.821±102243.407 | 0.943 | 73150.281±55867.847/  152139.821±102243.407 | 0.318 | 156686.992±53763.052/  134345.857±86667.744 | 0.695 | 73150.281±55867.847/  134345.857±86667.744 | 0.388 |
| 7-Lathosterol | 55.593(0.0-123.581)/  139.0(82.791-300.685) | 0.52 | 241.404±170.806/  181.848±126.370 | 0.504 | 68.689±46.926/  181.848±126.37 | 0.247 | 241.404±170.806/  82.396±98.405 | 0.093 | 68.689±46.926/  82.396±98.405 | 0.859 |

Table 3 SM. Statistical analyses of differences between the PKAN subgroups of metals. The data are presented as mean±SD or median(25%-75%) – depending on the test used of the PKAN patient subgroup vs control levels and between PKAN subgroups; for each analysis ANOVA test p values are presented with bold indicated significant differences. P1 – n=3, P2 – n=2, P3 – n=7, control n=12.

| Metal | P3 vs control [μM] | p | P1 vs control  [μM] | P | P2 vs control  [μM] | P | P1 vs P3  [μM] | p | P2 vs P3  [μM] | P |
| --- | --- | --- | --- | --- | --- | --- | --- | --- | --- | --- |
| Mg | 622.176±54.905/  706.628±72.401 | **0.015** | 691.331±45.726/  706.628±72.401 | 0.735 | 523.508±109.612/  706.628±72.401 | **0.007** | 691.331±45.726/  622.176±54.905 | 0.094 | 523.508±109.61/  622.176±54.905 | 0.103 |
| K | 3113.499±468.484/  3910.986±590.055 | **0.006** | 3124.346±250.39/  3910.986±590.055 | **0.044** | 2665.330±477.751/  3910.986±590.055 | **0.015** | 3124.346±250.395/  3113.499±468.484 | 0.971 | 2665.330±477.751/  3113.499±468.484 | 0.273 |
| Ca | 941.258±59.08/  1041.860±119.623 | 0.053 | 1045.173±66.218/  1041.860±119.623 | 0.964 | 836.628±189.441/  1041.860±119.623 | 0.052 | 1045.173±66.218/  941.258±59.08 | **0.039** | 836.628(702.673-970.583)/  950.553(882.343-993.616) | 0.500 |
| Cr | 5.716±0.609/  6.337±0.716 | 0.068 | 6.043±0.668/  6.337±0.716 | 0.527 | 4.652±1.627/  6.337±0.716 | **0.018** | 6.043±0.668/  5.716±0.609 | 0.469 | 4.652±1.627/  5.716±0.609 | 0.156 |
| Fe | 4.329(1.724-9.026)/  20.886(15.539-24.824) | **0.0001** | 18.609±9.737/  21.919±10.208 | 0.618 | 9.573(7.524-11.623)/  20.886(15.539-24.824) | 0.062 | 18.609±9.737/  5.315±4.023 | **0.012** | 9.573±2.898/  5.315±4.023 | 0.214 |
| Co | 0.0149(0.0119-0.022)/  0.00904(0.00791-0.0133) | **0.016** | 0.0115(0.00911-0.0134)/  0.00904(0.00791-0.0133) | 0.382 | 0.0186(0.0169-0.0202)/  0.00904(0.00791-0.0133) | 0.062 | 0.0113±0.00213/  0.0174±0.00707 | 0.192 | 0.0186±0.00235/  0.0174±0.00707 | 0.836 |
| Ni | 0.0929±0.0172/  0.0926±0.0402 | 0.985 | 0.111±0.00718/  0.0926±0.0402 | 0.453 | 0.0894±0.0202/  0.0926±0.0402 | 0.914 | 0.111± 0.00718/  0.0929±0.0172 | 0.125 | 0.0894±0.0202/  0.0929±0.0172 | 0.810 |
| Cu | 13.319(12.181-14.858)/  13.303(12.506-14.607) | 0.843 | 12.828(10.766-17.605)/  13.303(12.506-14.607) | 0.737 | 12.092±4.096/  13.915±2.496 | 0.382 | 13.733±3.508/  13.953±1.873 | 0.897 | 12.092±4.096/  13.953±1.873 | 0.351 |
| Zn | 2.968(2.358-3.604)/  5.936(5.390-9.787) | **0.001** | 4.442(4.15-5.655)/  5.936(5.390-9.787) | **0.037** | 5.039(3.796-6.281)/  5.936(5.390-9.787) | 0.308 | 4.749±0.798/  3.079±0.618 | **0.007** | 5.039(3.796-6.281)/  2.968(2.358-3.604 | 0.111 |
| Al | 2.822(1.988-5.437)/  7.200(5.712-11.374) | **0.008** | 12.482(11.122-17.361)/  7.200(5.712-11.374 | 0.122 | 8.907(6.061-11.753)/  7.200(5.712-11.374) | 0.734 | 13.655±3.281  3.813±2.016 | **0.001** | 8.907±4.025/  3.813±2.016 | **0.033** |
| B | 5.87(4.217-11.151)/  2.396(1.436-3.378) | **0.002** | 15.397(4.106-19.506)/  2.396(1.436-3.378) | **0.019** | 7.874(4.390-11.357)/  2.396(1.436-3.378) | 0.062 | 13.003±7.974/  7.354±3.866 | 0.155 | 7.874±4.926/  7.354±3.866 | 0.877 |
| Cd | 0.000414(0.000405-0.000531)/  0.000684(0.000373-0.00143) | 0.405 | 0.00141(0.00135-0.00164)/  0.000684(0.000373-0.00143) | 0.158 | 0.000576(0.000297-0.000855)/  0.000684(0.000373-0.00143) | 0.396 | 0.00141(0.00135-0.00164)/  0.000414(0.000405-0.000531) | **0.017** | 0.000576(0.000297-0.000855)/  0.000414(0.000405-0.000531 | 1.000 |
| Se | 0.792±0.169/  1.183±0.125 | **0.001** | 1.017(0.794-1.058)/  1.202(1.115-1.285) | **0.019** | 1.048(0.988-1.109)/  1.202(1.115-1.285) | 0.089 | 0.956±0.142/  0.792±0.169 | 0.179 | 1.048±0.0857/  0.792±0.169 | 0.084 |
| Mn | 0.368(0.266-0.577)/  0.0319(0.0237-0.04) | **0.013** | 0.402(0.3-0.461)/  0.0319(0.0237-0.04) | 0.069 | 0.363(0.253-0.473)/  0.0319(0.0237-0.04) | 0.126 | 0.388±0.0812/  0.401±0.170 | 0.903 | 0.363±0.156/  0.401±0.170 | 0.786 |
| Sr | 0.286±0.0529/  0.404±0.120 | **0.026** | 0.331±0.0575/  0.404±0.120 | 0.332 | 0.273±0.0148/  0.404±0.120 | 0.160 | 0.331±0.0575/  0.286±0.0529 | 0.270 | 0.273±0.0148/  0.286±0.0529 | 0.741 |

Table 4 SM. Instrumental settings and operating conditions for ICP-MS measurements.

| **Parameter** | | **Value** |
| --- | --- | --- |
| RF power | | 1300 W |
| Carrier gas flow (Ar) | | 0.88 L/min |
| Dwell time | | 50 ms |
| Readings | | 1 |
| Sweeps | | 5 |
| Monitored isotopes: | ^11^B, ^24^Mg, ^27^Al, ^39^K, ^43^Ca, ^53^Cr , ^55^Mn, ^57^Fe, ^59^Co, ^60^Ni, ^63^Cu, ^66^Zn, ^82^Se, ^88^Sr, ^112^Cd, | |
